# Cerebral Venous Outflow Insufficiency: A Study on Symptoms and Venous Stenosis Classification

**DOI:** 10.1002/mco2.70609

**Published:** 2026-02-26

**Authors:** Hui Li, Xiaojiao Guan, Lu Liu, Chunxiao Lu, Weiyue Zhang, Yifan Zhou, Huimin Jiang, Chenxia Zhou, Jian Dong, Xunming Ji, Chen Zhou

**Affiliations:** ^1^ Department of Neurology Xuanwu Hospital Capital Medical University Beijing China; ^2^ Beijing Institute of Brain Disorders Laboratory of Brain Disorders Ministry of Science and Technology Collaborative Innovation Center for Brain Disorders, Beijing Advanced Innovation Center for Big Data‐based Precision Medicine, Capital Medical University Beijing China; ^3^ Neuro Cardio Vascular Diseases Center Xuanwu Hospital Capital Medical University Beijing China; ^4^ Department of Radiology China‐Japan Friendship Hospital Beijing China; ^5^ Neuroscience Center Beijing Shijitan Hospital Capital Medical University Beijing China; ^6^ Beijing Advanced Innovation Center for Big Data‐Based Precision Medicine School of Biological Science and Medical Engineering Beihang University Beijing China; ^7^ Department of Radiology Beijing Tiantan Hospital Capital Medical University Beijing China

**Keywords:** cerebral venous outflow insufficiency, cerebral venous congestion symptoms, cerebral venous sinus stenosis, internal jugular venous stenosis, neuroimaging features

## Abstract

Cerebral venous outflow insufficiency (CVOI) is a recently recognized cerebrovascular condition characterized by impaired venous drainage from the brain to the extracranial system. However, its clinical phenotypes and classification criteria remain poorly defined. In this single‐center cross‐sectional study, we analyzed 245 patients with CVOI using contrast‐enhanced CT or MR venography to identify clinical features and propose a novel anatomical classification. We identified 10 major symptoms of cerebral venous congestion, with tinnitus cerebri, neck discomfort, and tinnitus being the most common. A new classification system was proposed based on lesion location and bilateral jugular foramen narrowing rate, categorizing CVOI into intracranial (CV), extracranial (JV), and tandem (CJV) types, each further stratified into four/five subtypes. Receiver operating characteristic (ROC) curve analysis showed that narrowing thresholds of 0.20 and 0.40 offered excellent discriminatory performance for subtype differentiation, with an area under the curve (AUC) approaching 1.0. Notably, tandem‐type CVOI (CJV) was the most prevalent (56.7%) and exhibited distinct symptom patterns and pathogenesis. These findings provide a practical framework for diagnosing and stratifying CVOI and may inform individualized treatment strategies.

## Graphical abstract

Cerebral venous outflow insufficiency (CVOI) predominantly consists of 10 cerebral venous congestion symptoms. Based on the traditional classification according to imaging features, this study classified CVOI into intracranial (CV), extracranial (JV), and tandem (CJV) types, which better reflect the imaging features and pathological types of patients to guide clinical management.

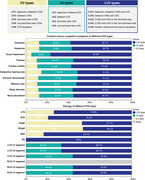

## 1 Introduction

The cerebral venous systems are integral to the intricate and dynamic interactions among intracranial compartments, involving the parenchyma, veins, arteries, and cerebrospinal fluid within the brain [1, 2, 3]. Each component plays a crucial role in maintaining intracranial homeostasis, collectively occupying space within the fixed volume of the calvarium. Similar to how ischemia indicates inadequate arterial inflow, congestion signifies insufficient venous outflow. Notably, cerebral venous outflow insufficiency (CVOI) is the primary cause of cerebral venous congestion [4].

Cerebral venous insufficiency has two major subtypes: cerebral venous sinus stenosis (CVSS) and internal jugular venous stenosis (IJVS). The internal jugular vein (IJV) is anatomically a continuation of the cerebral venous sinus (CVS). Several etiologies can lead to CVOI, such as intraluminal lesions (arachnoid granules (AGs), overgrown IJV valves, etc.), extraluminal compression (tumors, transverse process of the atlas, styloid process, etc.), and vessel wall lesions [5, 6]. However, previous studies of nonthrombotic CVOI have focused on CVSS or IJVS alone, neglecting patients with cerebrocervical venous tandem lesions and dysplasia [7, 8, 9, 10, 11, 12]. A significant proportion of patients have cerebrocervical venous tandem lesions [13, 14]. In addition, congenital developmental anomalies such as vascular dysplasia or defects are also important factors in impaired venous outflow [15]. In clinical practice, although elevated intraventricular pressure is an important factor in exacerbating CVOI, the pathogenesis of CVOI from different etiologies is not uniform and therefore has different imaging characteristics and benefits from different treatment modalities [16, 17, 18, 19, 20, 21, 22, 23, 24, 25, 26, 27]. Therefore, the available CVOI classifications are not yet a perfect guide to the appropriate management of all CVOI patients.

The traditional classification divides the normal population into balanced, dominant, and dysplastic types based on the morphology of the head and neck veins on imaging. Durgun et al. showed in 1993 that only 37.6% of the normal population had bilateral symmetry of the transverse sinus (TS) and sigmoid sinus (Sigs), whereas the majority was asymmetry, with a clear predominance of the right TS (RTS) and Sigs (61%) [28, 29]. In addition, approximately 68.5% of patients with IJV slenderness have ipsilateral TS and Sigs dysplasia or stenosis [30]. However, the traditional classification currently lacks clearly defined criteria, as the cerebrocervical venous system is characterized by a high degree of morphological variability and individual differences. In addition, the traditional classification has not yet been applied to the study of patients with CVOI. Regarding nonthrombotic CVOI, some studies have classified the population with cerebrocervical venous anomalies into two main types: cerebrocervical venous stenosis and dysplasia [30, 31, 32]. Characteristic imaging features of IJVS, including abnormal venous collaterals around the local stenosis, opaque white matter hyperintensity, and a mismatch between the transverse diameter of the IJV and the caliber of jugular foramen, have been found to distinguish it from physiological IJV slenderness [32]. And whether IJV slenderness leads to IJVS needs further study [32]. The specific subtypes of CVOI and the symptomatic and imaging characteristics of each type are still relatively confusing and undefined due to the nonspecific nature of CVOI symptoms and the lack of in‐depth knowledge of anatomical structures and associated imaging features.

Therefore, we analyzed the symptomatic and imaging characteristics of our single‐center patients with all types of CVOI using head and neck computed tomography (CT). Our study aimed to propose the main symptoms of CVOI, clarify the criteria for the traditional classification, and propose a new comprehensive classification of CVOI to guide clinical management.

## 2 Results

### 2.1 Relationship Between Traditional Cerebrocervical Venous Classification and Jugular Foramen Caliber

**FIGURE 1:**
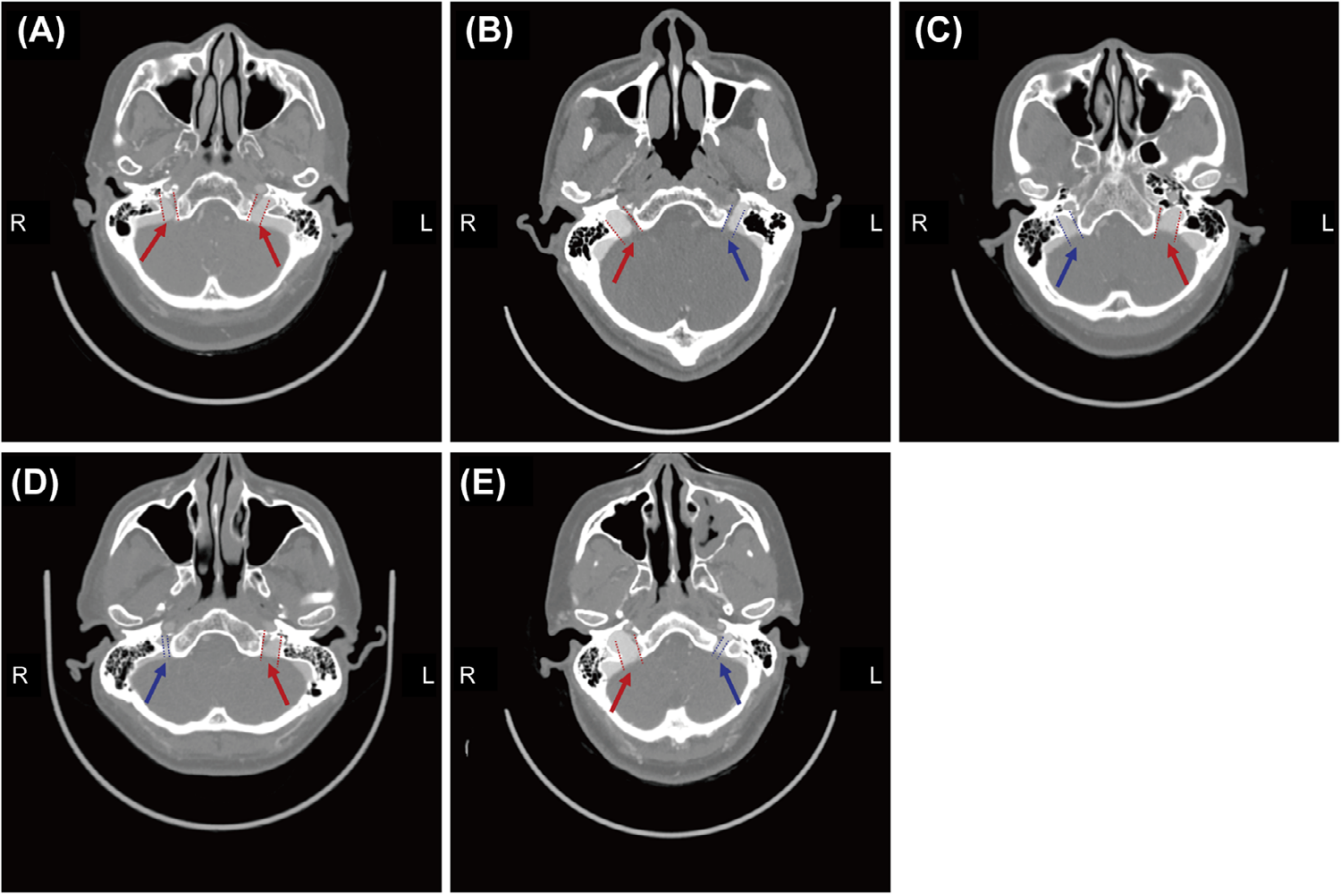
Modalities of the internal jugular foramen on CT (red and blue arrows): (A) balanced type, (B) right dominant type, (C) left dominant type, (D) right dysplastic type, and (E) left dysplastic type.

A total of 271 patients were initially enrolled in this study. Based on the bilateral symmetry of cerebrocervical venous anatomy, patients were categorized into three traditional morphological types: balanced (*n* = 115, 42.4%; Figure 1A), dominant (*n* = 66, 24.4%; Figure 1B,C), and dysplastic (*n* = 90, 33.2%; Figure 1D,E).

Differences in bilateral jugular foramen caliber and narrowing rate were calculated and compared among the groups (Tables S1 and S2). First, the dominant and dysplastic types were predominantly right dominant (*n* = 49, 74.2%) and left dysplastic (*n* = 69, 76.7%). In addition, there was no statistical difference in jugular foramen caliber diameter between the two sides of the balanced type (*p* > 0.05), whereas there was a significant difference between the two sides of the dominant and dysplastic types (*p* < 0.05). As shown in Table S1, the mean jugular foramen caliber diameter of the balanced type was 8.12 ± 0.99 mm on the left side and 8.14 ± 1.04 mm on the right side (*p* > 0.05). The mean jugular foramen caliber diameter in the right dominant type was 6.53 ± 0.90 mm on the left side and 9.27 ± 1.32 mm on the right side. The mean jugular foramen caliber diameter in the left dominant type was 8.99 ± 1.84 mm on the left side and 6.34 ± 1.29 mm on the right side. The mean diameter of the jugular foramen caliber in the right dysplastic type was 10.18 ± 1.54 mm on the left side and 5.04 ± 1.07 mm on the right side. The mean diameter of the jugular foramen caliber in the left dysplastic type was 4.44 ± 1.12 mm on the left side and 9.89 ± 1.63 mm on the right side. The minimum and maximum values were also shown in Table S1. The mean right jugular foramen diameter in males (9.96 ± 1.67 mm) was significantly higher than that in females in the right dominant type (8.90 ± 1.06 mm) (*p* < 0.05), while there was no statistical difference between genders in the other types (*p* > 0.05) (Table S2). Furthermore, the narrowing rate of the bilateral jugular foramen in the balanced, dominant, and dysplastic types was found to be 0–0.20, 0.20–0.40, and ≥0.40, respectively (Table S3), with no significant difference observed between left and right subtypes or between genders (*p* > 0.05) (Tables S3 and S4).

Receiver operating characteristic (ROC) curve analysis was conducted to identify optimal cutoff values for bilateral jugular foramen narrowing rate in classifying traditional CVOI subtypes. The analysis yielded two optimal thresholds: 0.203 for differentiating between balanced and dominant types and 0.395 for differentiating between dominant and dysplastic types (Figure S1A–D). These thresholds demonstrated excellent discriminatory performance. The area under the curve (AUC) for both approached 1.000, indicating perfect overall accuracy (Figure S1E,F). At the 0.203 threshold, the sensitivity and specificity were 0.994 and close to 1.000, respectively. At the 0.395 threshold, both sensitivity and specificity were nearly 1.000. Based on these results, we defined three ranges of narrowing rate, 0–0.20 (balanced), 0.20–0.40 (dominant), and ≥0.40 (dysplastic), which can serve as robust and objective criteria for classifying the three traditional subtypes of CVOI.

### 2.2 Cerebral Venous Congestion Symptoms and Imaging Features of CVOI

**FIGURE 2:**
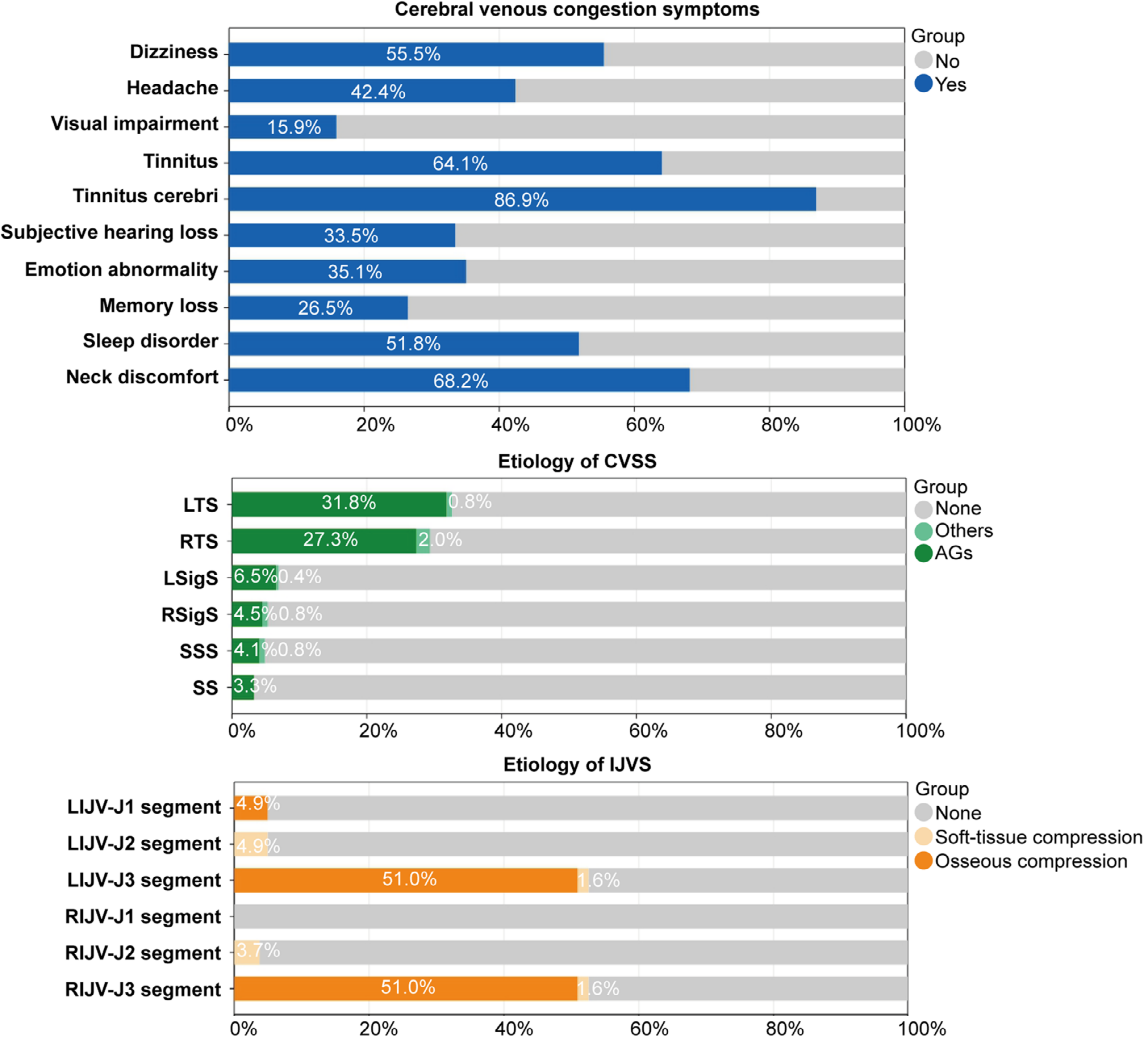
Cerebral venous congestion symptoms and etiology of CVOI. CVSS, cerebral venous sinus stenosis; IJVS, internal jugular vein stenosis; SSS, superior sagittal sinus; LTS, left transverse sinus; RTS, right transverse sinus; LSigS, left sigmoid sinus; RSigS, right sigmoid sinus; SS, straight sinus; AGs, arachnoid granules; LIJV, left internal jugular vein; RIJV, right internal jugular vein; CVOI, cerebral venous outflow insufficiency.

A total of 26 patients were excluded due to CVOI‐like symptoms being attributable to alternative etiologies. Based on imaging findings, 245 patients were ultimately diagnosed with CVOI and included in the subsequent analysis of clinical symptoms, imaging features, and pathological etiologies. Patients ranged in age from 24 to 86 (mean 53.34 ± 13.37) years with a male to female ratio of 118:127 (Table S5). As shown in Figure 2, the main symptoms of cerebral venous congestion in patients with CVOI included dizziness (*n* = 136, 55.5%), headache (*n* = 104, 42.4%), visual impairment (*n* = 39, 15.9%), tinnitus (*n* = 157, 64.1%), tinnitus cerebri (*n* = 213, 86.9%), subjective hearing loss (*n* = 82, 33.5%), emotional abnormality (*n* = 86, 35.1%), memory loss (*n* = 65, 26.5%), sleep disorder (*n* = 127, 51.8%), and neck discomfort (*n* = 167, 68.2%). Among these, tinnitus cerebri was the most common cerebral venous congestion symptom of CVOI. The pathological cause of CVSS was mainly AGs (94.06%) (Figure 2). There were also IJVS in all segments bilaterally, except for the right IJVS in segment J1 (Figure 2). IJVS in the left J1 segment was commonly due to compression of the sternum and aorta. IJVS in segment J3 was primarily due to osseous compression (transverse process of the atlas, styloid process, 96.90%) and a small proportion of soft‐tissue compression (internal carotid artery and digastric muscle, 3.10%).

### 2.3 Symptomatic and Pathostructural Analysis of Traditional Classification of CVOI

Symptoms, imaging features, and pathological causes were analyzed in 245 patients with CVOI. Based on the morphology of the bilateral cerebrocervical veins on imaging, CVOI was classified into three groups: balanced (*n* = 99, 40.4%), dominant (*n* = 56, 22.9%), and dysplastic (*n* = 90, 36.7%) types.

The three traditional types of CVOI also differed in demographic, symptomatic, and pathological characteristics (Table S5). CVOI was more common in the balanced and dysplastic types and least common in the dominant type. Females were significantly more prevalent in the dominant type than in the other two types (*p* < 0.05). Except for neck discomfort, which was most common in the dominant type (*p* < 0.05), the 10 main symptoms of CVOI did not differ significantly among the three types (*p* > 0.05). CVSS due to venous segmentation occurred predominantly in the dominant type, especially in the RTS (*p* < 0.05). The balanced type had the highest prevalence of IJVS (left: 66.7%, right: 72.7%, *p* < 0.05) and occurred predominantly in the J3 segment (left: 62.6%, right: 70.7%, *p* < 0.05) due to osseous compression. The dysplastic type was least frequent in the IJVS (left: 41.1%, right: 37.8%, *p* < 0.05), especially in the J3 segment (left: 37.8%, right: 38.9%, *p* < 0.05).

### 2.4 Symptomatic and Pathostructural Analysis of Simple CVS Outflow Deficiency

**FIGURE 3:**
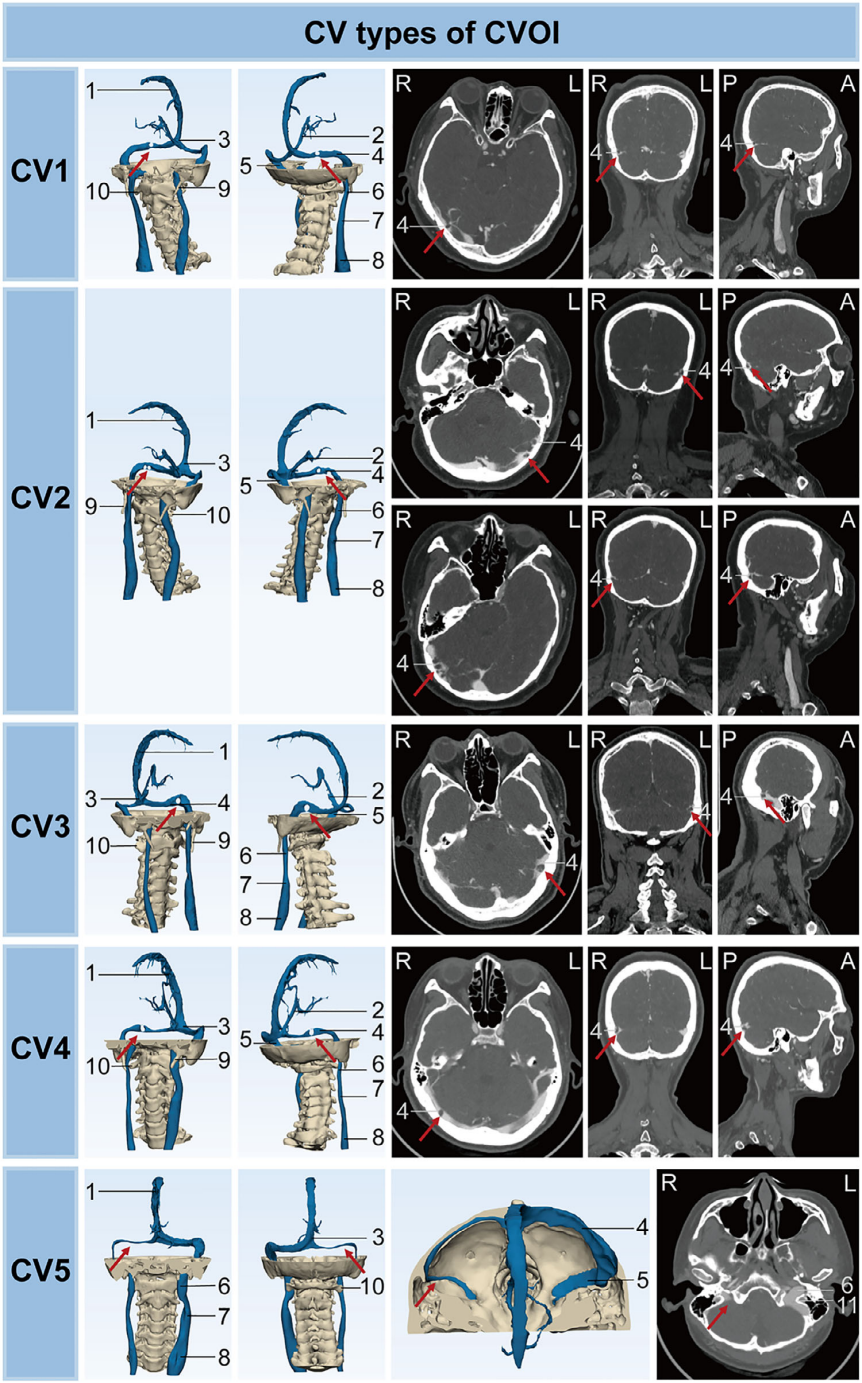
Imaging characteristics of CV types of CVOI on CT. Numbered labels: 1 = superior sagittal sinus, 2 = inferior sagittal sinus, 3 = confluence of sinuses, 4 = transverse sinus, 5 = sigmoid sinus, 6 = internal jugular vein (J3 segment), 7 = internal jugular vein (J2 segment), 8 = internal jugular vein (J1 segment), 9 = styloid process, 10 = transverse process of the atlas (C1), 11 = jugular foramen. CVOI, cerebral venous outflow insufficiency; CV, intracranial type of CVOI; R, right; L, left; P, posterior; A, anterior.

The symptoms, imaging features, and pathological causes of 29 patients with simple CVS outflow deficiency were analyzed (Table S6). Based on the morphology and pathological causes of the bilateral CVSs on imaging, intracranial (CV) type of CVOI was divided into five subtypes: CV1: balanced unilateral CVSS, CV2: bilateral CVSS (including bilateral CVSS, unilateral CVSS combined with contralateral dysplasia), CV3: dominant side CVSS, CV4: nondominant side CVSS, and CV5: CVS dysplasia (Figure 3). Of these, CV3 has the lowest proportion of CV types (*n* = 2, 6.9%). More than half of the patients with type CV developed tinnitus cerebri (*n* = 23, 79.3%), neck discomfort (*n* = 21, 72.4%), dizziness (*n* = 17, 58.6%), tinnitus (*n* = 16, 55.2%), and sleep disorder (*n* = 15, 51.7%). The percentage of dizziness was higher in type CV3 (*n* = 2, 100.0%) and CV4 (*n* = 8, 100.0%) compared with the other groups. In addition, type CV is more common in females (*n* = 21, 72.4%), especially type CV2 (*n* = 9, 90.0%). Furthermore, the main pathological cause of CVSS was AGs (96.3%), followed by venous septation, and so on. Type CV occurred most frequently in bilateral TS (left: 72.4%, right: 51.7%) and rarely in the straight sinus (SS). The proportion of stenosis in the bilateral TS and the occurrence of AGs in this location were higher in type CV2 than in the other groups (*p* < 0.05). Type CV5 also showed stenosis on the dysplastic side, predominantly bilateral in the TS and caused by AGs.

### 2.5 Symptomatic and Pathostructural Analysis of Simple IJV Outflow Deficiency

**FIGURE 4:**
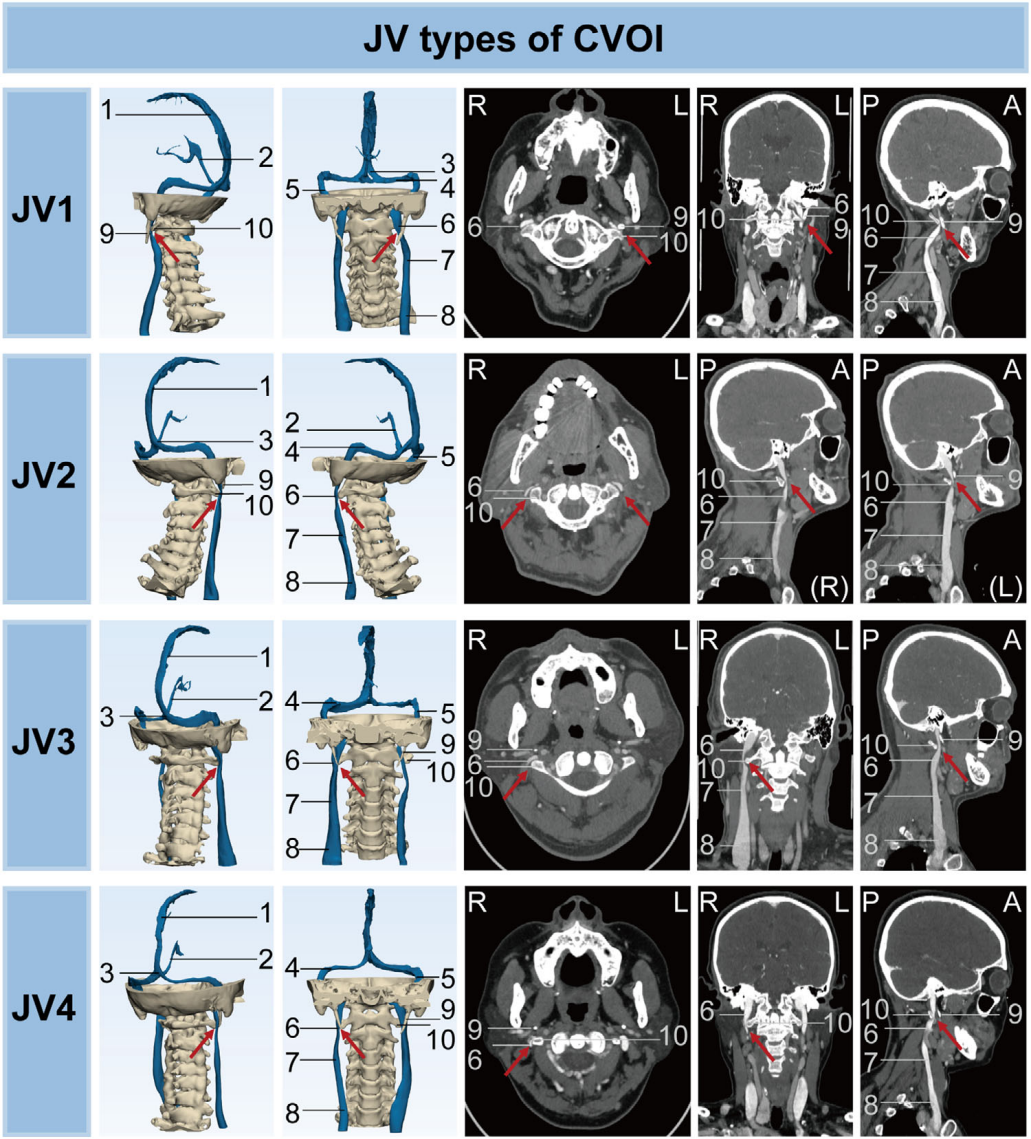
Imaging characteristics of JV types of CVOI on CT. Numbered labels: 1 = superior sagittal sinus, 2 = inferior sagittal sinus, 3 = confluence of sinuses, 4 = transverse sinus, 5 = sigmoid sinus, 6 = internal jugular vein (J3 segment), 7 = internal jugular vein (J2 segment), 8 = internal jugular vein (J1 segment), 9 = styloid process, 10 = transverse process of the atlas (C1). CVOI, cerebral venous outflow insufficiency; JV, extracranial type of CVOI; R, right; L, left; P, posterior; A, anterior.

The symptoms, imaging features, and pathological causes of 77 cases of simple IJV outflow deficiency were analyzed (Table S7). According to the morphology and pathological causes of the bilateral IJVs on imaging, the extracranial (JV) type of CVOI was divided into five subtypes: JV1: balanced unilateral IJVS, JV2: bilateral IJVS (including bilateral IJVS, unilateral IJVS combined with contralateral dysplasia), JV3: dominant IJVS, JV4: nondominant IJVS, and JV5: simple IJV dysplasia (Figure 4).

There were significant differences in imaging features and pathological causes among the different subtypes of JV types. First, no JV5 type was found in CVOI, probably because IJV dysplasia is usually associated with CVS dysplasia. The highest proportion of JV subtypes was found in type JV2 (*n* = 41, 53.2%), whose stenosis most commonly occurs in the bilateral J3 segments and was caused by osseous compression. As shown in Table S7, type JV3 was the rarest (*n* = 1, 1.3%). Type JV4 stenosis more commonly occurred in the left IJV, whereas type JV1 stenosis more commonly occurred in the right IJV. IJVS in the J1 segment almost always occurred on the left side and was caused by osseous compression (sternum and aorta, 100.0%). IJVS in the J2 segment was almost exclusively caused by soft‐tissue compression (sternocleidomastoid muscle and common carotid artery, 100.0%). In the JV type, IJVS in the J3 segment resulted primarily from osseous compression by the transverse process of the atlas and the styloid process (96.5%), and to a lesser extent from soft‐tissue compression (3.5%) by the internal carotid artery and the posterior belly of the digastric muscle. The digastric muscle, due to its anatomical course at the cranio‐cervical junction, may exert mechanical compression on the IJV, particularly at the J3 segment, and has been identified as a potential cause of external venous stenosis in previous studies [33].

There was no significant difference in symptoms among the JV subtypes (*p* > 0.05). Tinnitus cerebri (*n* = 68, 88.3%), neck discomfort (*n* = 54, 70.1%), tinnitus (*n* = 54, 70.1%), sleep disorder (*n* = 41, 53.2%), and dizziness (*n* = 40, 51.9%) were present in more than half of type JV cases. In addition, type JV was more common in males (*n* = 43, 55.8%), especially the type JV2 (*n* = 27, 65.9%).

### 2.6 Symptomatic and Pathostructural Analysis of Cerebrocervical Venous Tandem Stenosis

**FIGURE 5:**
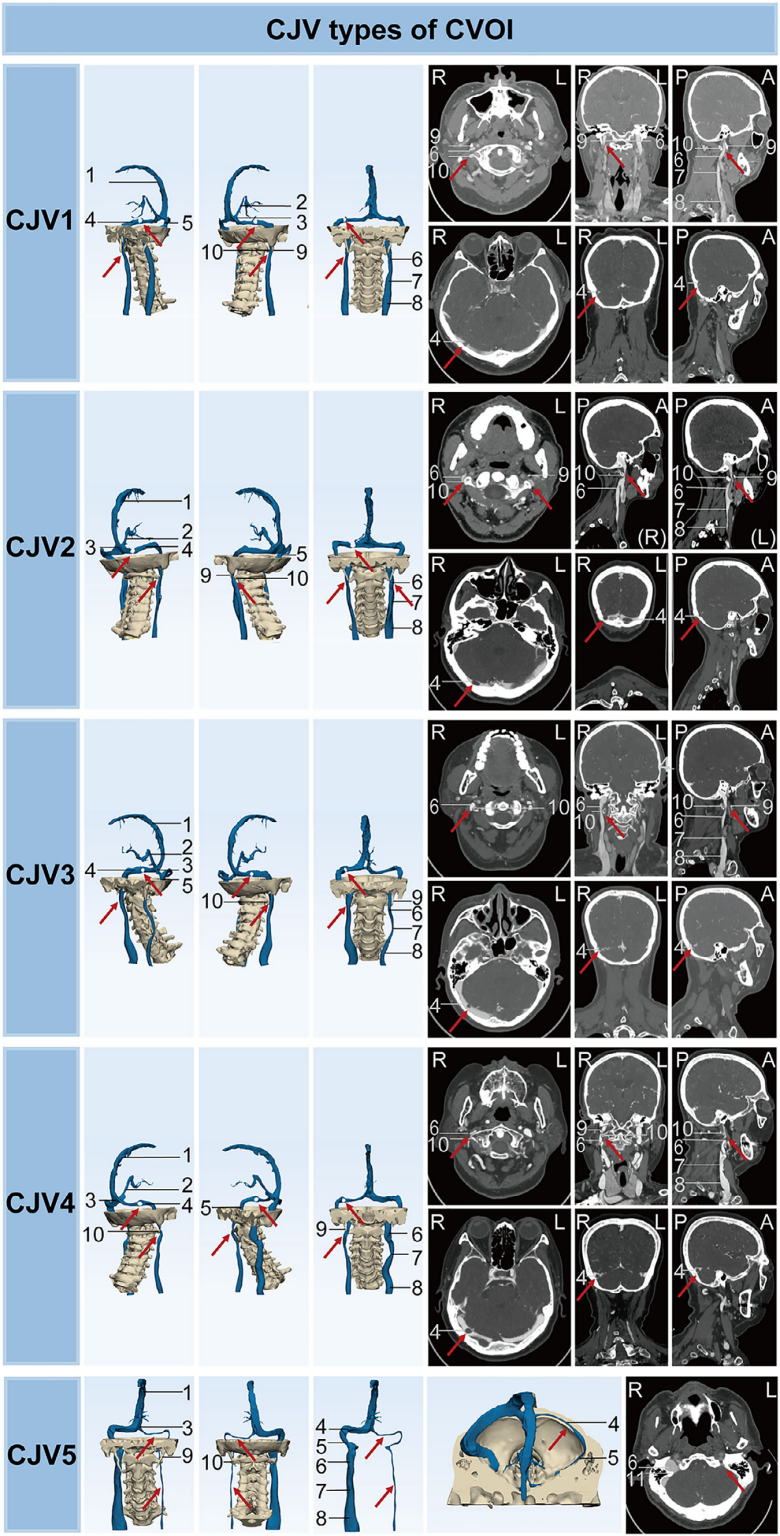
Imaging characteristics of CJV types of CVOI on CT. Numbered labels: 1 = superior sagittal sinus, 2 = inferior sagittal sinus, 3 = confluence of sinuses, 4 = transverse sinus, 5 = sigmoid sinus, 6 = internal jugular vein (J3 segment), 7 = internal jugular vein (J2 segment), 8 = internal jugular vein (J1 segment), 9 = styloid process, 10 = transverse process of the atlas (C1), 11 = jugular foramen. CVOI, cerebral venous outflow insufficiency; CJV, intracranial and extracranial tandem type of CVOI; R, right; L, left; P, posterior; A, anterior.

The symptoms, imaging characteristics, and pathological causes of 139 cases with cerebrocervical venous tandem stenosis were analyzed (Table S8). The tandem (CJV) type of CVOI was divided into five subtypes according to the morphology and pathological causes of the bilateral CVSs and IJVs on imaging: CJV1: balanced unilateral CVSS and IJVS, CJV2: bilateral CVSS and IJVS (including bilateral CVSS and IJVS, bilateral CVSS/IJVS, and dysplasia), CJV3: CVSS and IJVS on the dominant side, CJV4: CVSS and IJVS on the nondominant side, and CJV5: tandem cerebrocervical venous dysplasia (Figure 5). As shown in Table S8, stenosis of the superior sagittal sinus was most common in CJV4 (*p* < 0.05). Of the CJV subtypes, CJV2 was the most common (*n* = 89, 64.0%) and CJV3 was the rarest (*n* = 1, 0.7%). CJV2 was most often associated with bilateral TS stenosis (TSS) and bilateral J3 segment stenosis, which were mainly caused by AGs and osseous compression, respectively. In addition, IJVS in the J1 and J2 segments also occurred predominantly in the CJV2 type. Tinnitus cerebri (*n* = 122, 87.8%), neck discomfort (*n* = 92, 66.2%), tinnitus (*n* = 87, 62.6%), dizziness (*n* = 79, 56.8%), and sleep disorder (*n* = 71, 51.1%) were present in more than half of the CJV types. Visual impairment occurs in more than one fifth of CJV patients (*n* = 30, 21.6%).

### 2.7 CVOI Classification Proposed in this Study

**FIGURE 6:**
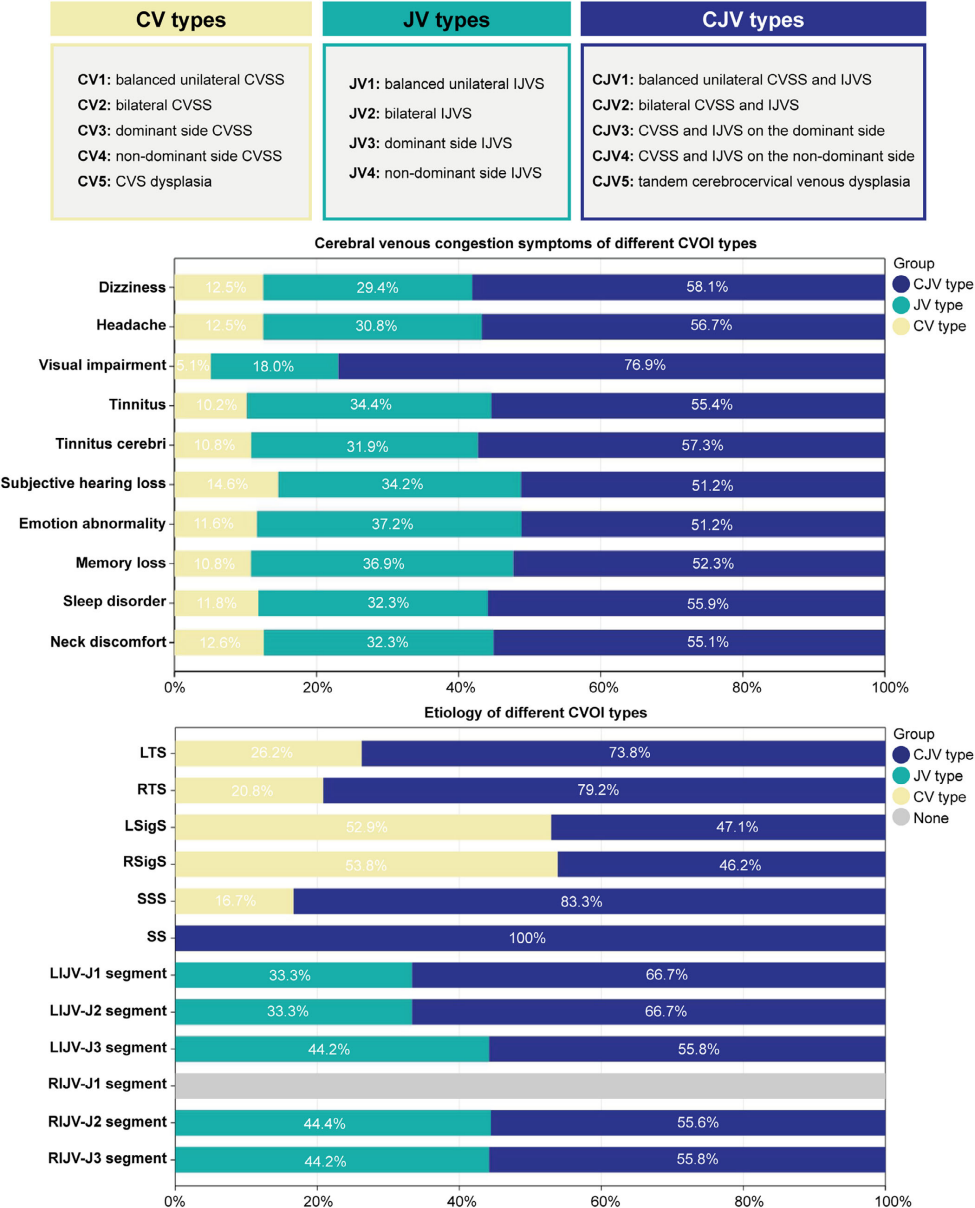
Criteria for defining new subtypes of CVOI and symptoms and etiology of cerebral venous congestion in different CVOI types. CVSS, cerebral venous sinus stenosis; IJVS, internal jugular vein stenosis; SSS, superior sagittal sinus; LTS, left transverse sinus; RTS, right transverse sinus; LSigS, left sigmoid sinus; RSigS, right sigmoid sinus; SS, straight sinus; AGs, arachnoid granules; LIJV, left internal jugular vein; RIJV, right internal jugular vein; CVOI, cerebral venous outflow insufficiency; CV, intracranial type of CVOI; JV, extracranial type of CVOI; CJV, intracranial and extracranial tandem type of CVOI.

**FIGURE 7:**
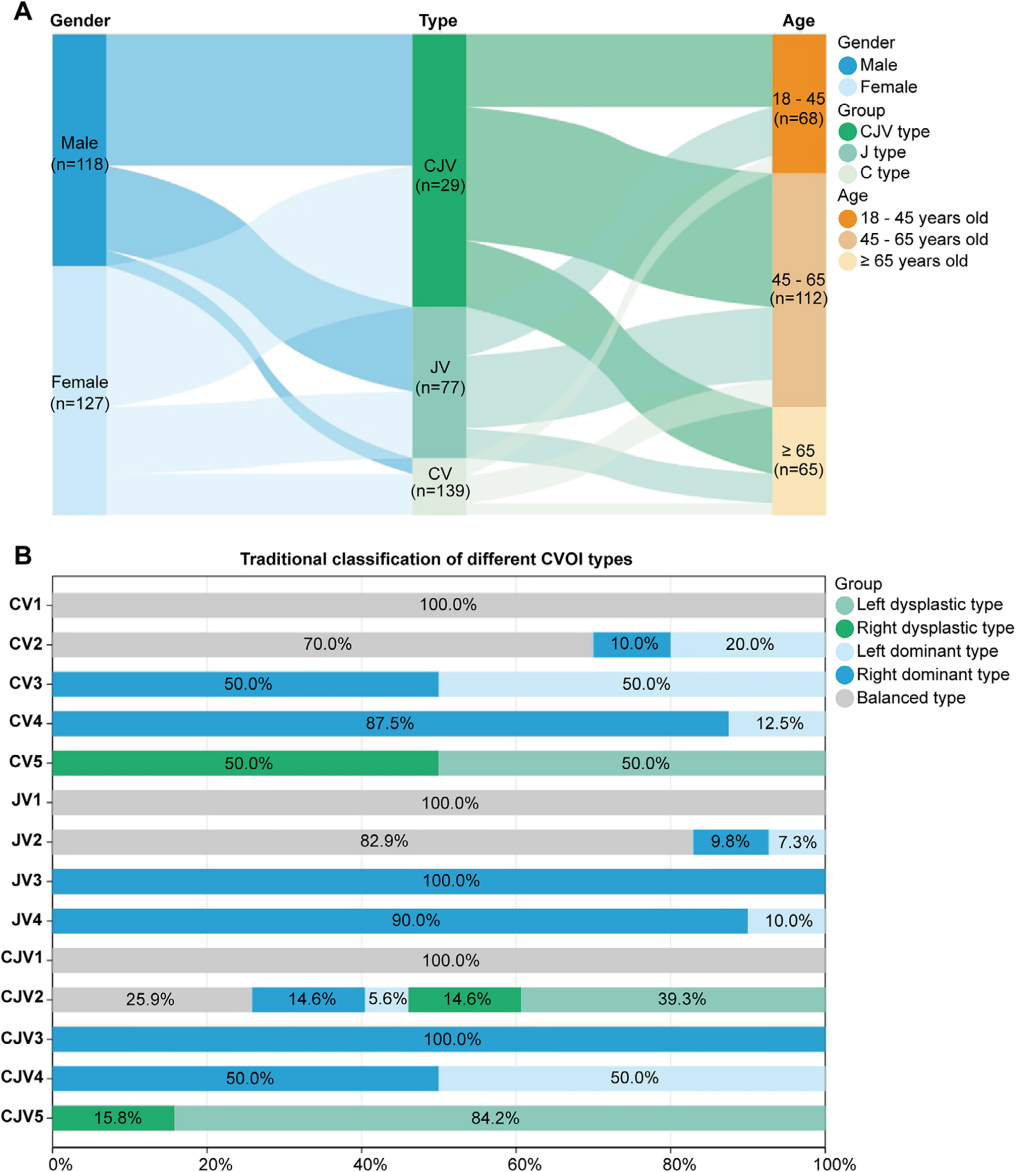
(A) Demographic characteristics of the CV, JV, and CJV types of CVOI; (B) traditional classification of the different types of CVOI. CVOI, cerebral venous outflow insufficiency; CV, intracranial type of CVOI; JV, extracranial type of CVOI; CJV, intracranial and extracranial tandem type of CVOI.

The symptoms, imaging characteristics and pathological causes of 245 cases of CVOI were analyzed (Figure 6 and Table S9). Based on the morphology of the bilateral cerebrocervical veins on imaging and the pathological causes, CVOI was classified into three main types: type CV, type JV, and type CJV. The symptoms and imaging features of the three types differed significantly. Among the three types, type CJV had the highest prevalence (*n* = 139, 56.7%), followed by type JV (*n* = 77, 31.4%), and type CV had the lowest prevalence (*n* = 29, 11.8%). As shown in Table S9 and Figure 7A, there was no significant gender difference among CVOI cases as a whole. However, type CV was more common in females (*n* = 21, 72.4%) and type JV was more common in males (*n* = 43, 55.8%). And the CJV subtype showed an approximately balanced sex distribution, with females comprising 51.8% of cases (*n* = 72). Patients were predominantly aged 45–65 years overall and in all three types. Furthermore, tinnitus cerebri, neck discomfort, tinnitus, dizziness, and sleep disorder occurred in more than half of patients in all three types, with tinnitus cerebri being particularly prevalent. Visual impairment occurred mainly in type CJV (*n* = 30, 21.6%). Dysplasia type was only present in types CV and CJV, and cerebrocervical venous tandem dysplasia was the predominant dysplasia type in CVOI (*n* = 38, 90.5%) (Tables S6–S8).

As shown in Figure 7B, the CV2 and JV2 types were predominantly balanced, whereas the CJV2 types were predominantly dysplastic, especially dysplastic on the left side. CV2, JV2, and CJV2 were the most common subtypes of CV, JV, and CJV types, whereas CV3, JV3, and CJV3 were the rarest. CV4 and JV4 were both predominantly left dominant types, whereas CJV4 occurred equally in right and left dominant types. CJV5 was predominantly the left‐sided dysplasia, whereas type CV5 occurred equally in right‐ and left‐sided dysplasia. Type CJV stenosis was most common in bilateral TS and bilateral J3 segments and was mainly caused by AGs or osseous compression, which was consistent with types CV and JV (Tables S6–S8). However, SS stenosis rarely occurred in CV type and was more common in CJV type, especially in CJV2 and CJV5 (Figure 6 and Table S8).

## 3 Discussion

CVOI is an emerging cerebrovascular disorder characterized by impaired drainage from the brain to the extracranial venous system [1, 2, 3]. Despite growing clinical awareness, its pathophysiological mechanisms, diagnostic criteria, and classification systems remain undefined [16, 17, 18, 19, 20, 21, 22, 23, 24, 25, 26, 27, 34]. Previous studies have primarily focused on isolated lesions such as CVSS or IJVS, often overlooking tandem or composite venous pathologies that may account for a broader spectrum of clinical symptoms. Furthermore, the heterogeneity of venous anatomy and the absence of standardized classification frameworks pose challenges for accurate diagnosis and patient stratification.

In this study, we systematically analyzed the clinical presentations and imaging features of 245 patients with CVOI. Ten major symptoms of cerebral venous congestion were identified, with tinnitus cerebri, neck discomfort, and tinnitus being the most prevalent. Imaging analysis revealed that stenoses in CV‐type patients were predominantly located in the bilateral TSs and Sigs, frequently accompanied by AGs. The co‐occurrence of AGs and morphological alterations such as Sigs wall dehiscence or diverticulum has been reported in the literature and may be relevant to the clinical manifestation of pulsatile tinnitus [35, 36, 37, 38, 39]. In contrast, stenosis of the SS was more commonly observed in CJV‐type patients. From an etiological perspective, IJVS was observed across multiple cervical segments in CVOI patients, with the exception of the right J1 segment. The pathogenesis of IJVS varied according to cervical segment: J2 segment stenosis was primarily attributed to soft‐tissue compression (e.g., sternocleidomastoid muscle, common carotid artery), whereas J3 segment stenosis was predominantly due to osseous compression (e.g., styloid process, transverse process of the atlas) [40, 41, 42]. Notably, bilateral IJVS and venous dysplasia were frequently present and often coexisted in CVOI patients. These findings indicate that analyses limited to isolated stenotic segments might not fully reflect the structural complexity commonly observed in patients with CVOI.

To address this complexity, we proposed a novel classification system that integrates traditional venous dominance patterns, narrowing rate of bilateral jugular foramen, and lesion location within the cerebrocervical venous axis. CVOI was categorized into three major types: CV, JV, and CJV, and each type was further stratified into four/five subtypes based on symmetry, dominance, and developmental anomalies. Among them, CJV was the most prevalent (56.7%), highlighting the clinical significance of tandem lesions. We also proposed novel diagnostic thresholds for the narrowing rate of bilateral jugular foramen (0–0.20 for balanced, 0.20–0.40 for dominant, and ≥0.40 for dysplastic), which demonstrated strong alignment with traditional morphological subtypes and may serve as objective reference points for future diagnosis and subtype differentiation.

Further stratified analysis revealed marked differences in demographic profiles, symptom distribution, imaging characteristics, and etiological mechanisms among the CV, JV, and CJV types. Bilateral lesions (CV2, JV2, CJV2) were the most common subtypes within each group, likely due to reduced compensatory capacity from the contralateral venous pathway, leading to more severe symptomatology. In contrast, dominant‐side lesions (CV3, JV3, CJV3) were relatively rare, potentially due to the structural and functional robustness of dominant venous outflow pathways. Although the overall sex distribution did not differ significantly, CV‐type CVOI (especially CV2) was more common in females, whereas JV‐type (particularly JV2) was more prevalent in males. Across all types, more than half of the patients presented with tinnitus cerebri, neck discomfort, tinnitus, dizziness, and sleep disorder. Visual impairment was most prominent in the CJV type, and may progress rapidly, severely impacting quality of life if left unrecognized.

Regarding venous dysplasia, previous studies have proposed that long‐segment narrowing exceeding 40% compared with the contralateral side should be classified as dysplasia [43, 44]. Our findings support this definition and further demonstrate that patients with isolated venous dysplasia, without evident focal stenosis, can still present with typical CVOI symptoms. Therefore, developmental anomalies should be included in the CVOI diagnostic spectrum. Notably, the JV5 subtype was not observed in this cohort; this absence coincided with a high prevalence of concurrent IJV and CVS dysplasia, which may reflect overlapping anatomical patterns [30]. Consistently, dysplastic changes were predominantly observed in the CV and CJV types, with tandem cerebrocervical venous dysplasia accounting for 90.5% of all dysplastic cases.

This study has several clinical implications. First, venous etiology should be considered in patients presenting with unexplained symptoms such as dizziness, tinnitus, visual impairment, and sleep disorder, symptoms that are frequently overlooked or misattributed. Second, a high prevalence of tandem lesions in the CJV subtype was observed among patients whose prior management targeted only one site, either CVSS or IJVS, rather than the entire cerebrocervical venous axis. This co‐distribution pattern underscores the potential value of comprehensive assessment and management across the whole axis. Third, the proposed classification system offers a practical framework for future clinical investigations and supports individualized treatment strategies based on anatomical subtype and etiology.

Nonetheless, this study has several limitations. First, although our diagnostic workflow incorporated color Doppler ultrasonography in multiple head and neck positions to reduce the risk of overlooking posture‐dependent IJVS, other dynamic or invasive imaging techniques, such as intravascular ultrasound, digital subtraction angiography (DSA), and retrograde pressure gradient measurement, were not systematically employed due to equipment limitations, institutional protocols, inherent procedural risks (e.g., hemorrhage, infection), and patients’ preference for conservative management [33, 45, 46, 47, 48, 49, 50]. As a result, some dynamically apparent but functionally significant lesions may have been underdiagnosed, and the dataset for invasive assessments was incomplete. Second, all cross‐sectional imaging (computerized tomography venography [CTV] and contrast‐enhanced magnetic resonance venography [CE‐MRV]) was performed in the supine, neutral head position, which may have limited the detection of transient or posture‐dependent venous compressions. Third, physiological confirmation, such as symptomatic relief following lumbar puncture, acetazolamide treatment, or venous interventions, was not consistently available, particularly in patients who declined invasive procedures. Fourth, this was a single‐center study with prospective data collection, which may limit the generalizability of our findings. Finally, because the analyses were cross‐sectional, causal relationships between anatomical phenotypes and clinical symptoms cannot be inferred, and the observed associations should be interpreted with caution. Future prospective, multicenter studies incorporating dynamic imaging techniques, physiological validation, and treatment outcomes are warranted to further refine the classification framework and evaluate the clinical relevance of CVOI.

## 4 Methods and Statistical Analysis

### 4.1 Patient Enrollment

Eligible patients were enrolled in this single‐center cross‐sectional study from June 1, 2023 to January 1, 2025 after obtaining signed informed consent and institutional ethics committee approval (Beijing Shijitan Hospital, Capital Medical University). All patients underwent contrast‐enhanced head and neck CTV or CE‐MRV at our hospital due to clinical suspicion of CVOI. Eligible patients were classified into balanced, dominant, and dysplastic types based on the bilateral symmetry of their cerebrocervical veins, as assessed by specialized imaging specialists. To avoid enrolling asymptomatic individuals with incidental anatomical variations, only patients with persistent symptoms of CVOI and radiologically confirmed venous stenosis (CVSS and/or IJVS) based on established imaging criteria were included as CVOI cases for further analyses.

Inclusion criteria:

1. Patients undergoing head and neck CTV/CE‐MRV in our hospital;
2. Patients presenting with typical symptoms related to CVOI;
3. Age ranged from 18 to 80 years;
4. No gender preference.

Exclusion criteria:

1. Patients with acute intracranial or carotid artery disease, including recent ischemic stroke, hemorrhage, or dissection, and those with congenital vascular malformations (e.g., arteriovenous malformations, cavernous malformations, developmental venous anomalies, or telangiectasias), due to their potential to confound hemodynamic evaluation or mimic CVOI‐related symptoms;
2. All subtypes of cerebral venous thrombosis;
3. Intracranial malignant tumors;
4. Severe hepatic or renal insufficiency and intolerance to CT or magnetic resonance imaging (MRI) due to known disease;
5. Contraindications for enhanced CT/MRI;
6. Patients who refused to sign informed consent or did not complete CTV/CE‐MRV;
7. History of head and neck cancer and previous head and neck surgery;
8. Patients with CVOI‐like symptoms that could be explained by other causes.

### 4.2 Baseline Information Collection

The primary symptoms associated with CVOI were recorded, including dizziness, headache, visual impairment, tinnitus, tinnitus cerebri, subjective hearing loss, emotion abnormality, memory loss, sleep disorder, and neck discomfort. Age, sex, and relevant medical history such as cerebrovascular disease, hypertension, diabetes mellitus, dyslipidemia, and cardiovascular event were also recorded. All cases meeting the inclusion criteria were included in the final analysis.

### 4.3 Imaging Evaluation and Diagnostic Classification

Imaging data of enrolled patients were retrospectively retrieved from inpatient and outpatient radiological databases. All patients underwent routine contrast‐enhanced head and neck CTV/CE‐MRV examinations. Two experienced neuroradiologists independently reviewed the CTV and CE‐MRV images. For patients who had undergone venous sinus stenting, IJV stenting, or IJV decompression surgery, additional imaging modalities such as DSA were also reviewed. Each neuroradiologist independently assessed the morphology of the bilateral CVSs and IJVs and categorized them into one of three traditional symmetry‐based subtypes: balanced, dominant, and dysplastic, based on CTV or CE‐MRV findings. Subsequently, diameters of the bilateral jugular foramen calibers were measured and compared among the three groups.

In this study, patients presenting with typical symptoms of CVOI were diagnosed with CVOI only if radiological evidence of CVSS and/or IJVS was confirmed on head and neck CTV, CE‐MRV, or DSA. The diagnostic workflow for CVOI used in this study is illustrated in Figure S2 [49, 50, 51, 52, 53, 54, 55, 56, 57]. The diagnostic criteria for IJVS included the following: (1) Focal IJV stenosis: Defined as a ≥50% reduction in diameter at the narrowest segment of the IJV compared with the adjacent upstream segment on the same imaging plane (axial or sagittal) as visualized on CE‐MRV, CTV, or DSA. In addition to the degree of narrowing, the presence of prominent collateral venous pathways, such as paraspinal or vertebral venous plexuses, was required to confirm the hemodynamic significance of the lesion. Morphologically, the stenotic segment typically demonstrated a characteristic “hourglass‐like” configuration [32, 33, 58, 59]. (2) IJV dysplasia: Characterized by hypoplastic IJV, often accompanied by narrowing of the ipsilateral jugular foramen compared with the contralateral side [32]. CVSS was diagnosed based on the following criteria: (1) Focal luminal narrowing: Presence of a ≥50% reduction in diameter at the site of stenosis compared with an adjacent normal segment on the same plane, or visible filling defects on CE‐MRV, CTV, or DSA [60, 61]. In addition to quantitative criteria, several qualitative features of stenosis were also considered, including focal collapse of the sinus wall, absence of a definable sinus lumen on one or more contiguous slices, and proximal poststenotic dilatation [62]. (2) CVS dysplasia: Defined as hypoplasia of one or both TSs, based on significantly reduced caliber or absence of flow‐related signal. All CVOI patients were categorized into one of three etiological subtypes based on the morphology and etiology of CVS and IJV abnormalities: type CV (CV1–CV5), type JV (JV1–JV5), and type CJV (CJV1–CJV5). The types of pathological stenosis and the associated clinical symptoms were recorded and compared across these classification groups.

### 4.4 Statistical Analysis

Continuous variables were tested for conformity to normal distribution using the Kolmogorov–Smimov test, and conformity to normal distribution was expressed as mean ± standard deviation (mean ± SD). The independent samples *t*‐test was used for comparisons between the two groups. Comparisons between multiple groups were made using one‐way analysis of variance (normally distributed and chi‐squared), and two by two comparisons between groups were made using the least significant difference method. When continuous variables were not normally distributed, the Mann–Whitney *U* test was used for between‐group comparisons. When continuous variables were not normally distributed or the variance was not homogeneous, comparisons between multiple groups were made using the Kruskal–Wallis *H* test, and two by two comparisons of the Kruskal–Wallis test were made using the Mann–Whitney *U* test with Bonferroni correction. Categorical variables were statistically described as numbers and percentages, and comparisons between groups were made using the chi‐squared or Fisher exact test. Two by two comparisons between groups were made using the Bonferroni correction. A two‐tailed *p* value of less than 0.05 was considered statistically significant. All statistical analyses were performed with SPSS version 26.0 for Windows. ROC curve analysis was performed using R software (version 4.5.1) to determine the optimal cutoff values for bilateral jugular foramen narrowing rates used to distinguish among the balanced, dominant, and dysplastic types. The AUC, sensitivity, specificity, and Youden index were calculated to assess diagnostic performance. The optimal thresholds were selected based on the maximum Youden index.

## 5 Conclusion

CVOI is a complex and often under‐recognized cerebrovascular disorder, with nonspecific symptoms and no established diagnostic standard, making clinical identification and treatment challenging. In this study, we systematically characterized the major symptoms of cerebral venous congestion and, for the first time, proposed an imaging‐based classification system that integrates traditional venous morphology with bilateral jugular foramen narrowing rates. The proposed subtypes, CV, JV, and CJV, reflect the anatomical and etiological heterogeneity of CVOI and provide a practical framework for clinical evaluation and individualized management. While our findings suggest the potential utility of this classification system, several limitations must be acknowledged. These include the lack of posture‐dependent dynamic imaging and direct hemodynamic assessments, the single‐center nature of the study, and the relatively small sample size in certain subgroups, which may limit statistical power. Therefore, further validation in large‐scale, multicenter, prospective studies is warranted to confirm the diagnostic and prognostic value of the proposed classification. Clinicians should consider CVOI in patients presenting with unexplained symptoms such as dizziness, headache, tinnitus, or visual impairment. Comprehensive imaging of the cerebrocervical venous drainage system may aid in early recognition, classification, and targeted intervention, ultimately improving patient outcomes.

## Author Contributions

Hui Li, Xiaojiao Guan, Lu Liu, Chunxiao Lu, Weiyue Zhang, Yifan Zhou, Huimin Jiang, Chenxia Zhou, Jian Dong, Xunming Ji, and Chen Zhou conceived and designed the study. Hui Li, Xiaojiao Guan, Lu Liu, and Chunxiao Lu contributed to data acquisition, analysis, or interpretation. Lu Liu, Hui Li, and Xiaojiao Guan drafted the manuscript. Jian Dong and Chen Zhou critically revised the manuscript for important intellectual content. Hui Li, Xiaojiao Guan, Lu Liu, Weiyue Zhang, and Yifan Zhou performed the statistical analysis. Huimin Jiang, Chenxia Zhou, and Chunxiao Lu provided administrative, technical, or material support. Chen Zhou, Jian Dong, and Xunming Ji supervised the study. All authors have read and approved the final manuscript.

## Conflicts of Interest

The authors declare no conflicts of interest.

## Ethics Statement

This study was approved by the Ethics Committee of Beijing Shijitan Hospital, Capital Medical University (Ethics Approval Number: sjtky11‐1x‐2022(013)) and was conducted in accordance with the ethical principles of the Declaration of Helsinki. Additionally, this study has been registered in the Chinese Clinical Trial Registry (ChiCTR) (Registration Number: ChiCTR2300071417).

## Consent

All participants voluntarily participated in this study after providing informed consent and signing a written informed consent form.

## Supporting information




**Table S1**: Comparison of the caliber of jugular foramen (right vs. left).
**Table S2**: Comparison of the caliber of jugular foramen (female vs. male).
**Table S3**: Comparison of bilateral jugular foramen caliber narrowing rates (right‐type vs. left‐type).
Table S4. Comparison of bilateral jugular foramen caliber narrowing rates (females vs. males).
**Table S5**: Demographic data, symptomatic data, and imaging features of CVOI with traditional classification.
**Table S6**: Demographic data, symptomatic data, and imaging features of CV type CVOI.
**Table S7**: Demographic data, symptomatic data, and imaging features of JV type CVOI.
**Table S8**: Demographic data, symptomatic data, and imaging features of CJV type CVOI.
**Table S9**: Demographic and symptomatic data of newly proposed CVOI classification.
**Figure S1**: Determination of optimal thresholds of narrowing rate in bilateral jugular foramen caliber and their predictive performance for imaging‐based classification. (A and B) Histograms showing the distribution of narrowing rates in the bilateral jugular foramen caliber and classification based on optimal cut‐off values. The best cut‐off points determined by the Youden index were 0.203 (A) and 0.395 (B), used to stratify subjects into different narrowing rate groups. (C and D) Curves of Youden index across a range of narrowing rate thresholds. The maximum Youden index indicated the optimal cut‐off values of 0.203 (C) and 0.395 (D), respectively. (E and F) ROC curves evaluating the predictive performance of narrowing rate thresholds (0.203 and 0.395) for imaging‐based classification. Both thresholds demonstrated excellent discrimination, with AUC values of 1.000. ROC, receiver operating characteristic; AUC, area under the curve.
**Figure S2**: Standardized diagnostic workflow for patients with CVOI. This flowchart outlines the standardized diagnostic approach employed in this study for patients with suspected CVOI‐related symptoms. The workflow begins with a multidisciplinary clinical evaluation (including neurology, ophthalmology, otolaryngology, spine surgery, and endocrinology) to exclude alternative systemic etiologies. Subsequent multimodal neuroimaging includes MRA/CTA to rule out intracranial and extracranial arterial pathologies, conventional MRI and CE‐MRI to assess structural brain lesions, and BB‐MRI to exclude acute or chronic cerebral venous sinus thrombosis. Doppler ultrasound assessments incorporate TCD, CADU, and CDUS‐IJV. The latter includes dynamic head‐rotation maneuvers (“head‐turning test”) to evaluate posture‐dependent IJVS, especially at the J3 segment, with systematic evaluation of J1‐J3 segments regarding vessel caliber, flow velocity, volume, waveform, and valvular function. In cases suggestive of intracranial hypertension (e.g., headache or visual disturbances), neuro‐ophthalmological assessments are performed, including visual acuity and field testing, funduscopy, OCT to quantify RNFL thickness, and ocular ultrasonography to measure ONSD. Noninvasive ICP monitoring or lumbar puncture for direct ICP measurement is performed when indicated. For patients meeting interventional criteria, DSA is conducted, followed by individualized treatment strategies such as venous sinus stenting, internal jugular vein decompression, optic nerve sheath fenestration, acetazolamide therapy, anticoagulation, weight reduction, and symptomatic management. CVOI, cerebral venous outflow insufficiency; CTA, computed tomography angiography; MRA, magnetic resonance angiography; MRI, magnetic resonance imaging; CE‐MRI, contrast‐enhanced magnetic resonance imaging; BB‐MRI, Black‐Blood MRI; CE‐CTV, contrast‐enhanced CT venography; CE‐MRV, contrast‐enhanced magnetic resonance venography; DSA, digital subtraction angiography; ICP, intracranial pressure; OCT, optical coherence tomography; RNFL, retinal nerve fiber layer; ONSD, optic nerve sheath diameter; IJVS, internal jugular vein stenosis; IJV, internal jugular vein, CDUS‐IJV, color Doppler ultrasonography of the internal jugular vein; TCD, Transcranial Doppler; CADU, carotid artery duplex ultrasonography.

## Data Availability

The authors have nothing to report.
