# Supplementary material for: Cerebral Venous Outflow Insufficiency: A Study on Symptoms and Venous Stenosis Classification
Source: MedComm (2020). 2026 Feb 26;7(3):e70609. doi: 10.1002/mco2.70609 (PMC12946657; doi:10.1002/mco2.70609)

**Cerebral Venous Outflow Insufficiency: A Study on Symptoms and Venous Stenosis Classification**

Hui Li^1, 2, 3, #^, Xiaojiao Guan^4, 5, #^, Lu Liu^1, 3^, Chunxiao Lu^1^, Weiyue Zhang^6^, Yifan Zhou^2^, Huimin Jiang^2^, Chenxia Zhou^1^, Jian Dong^7, 5, *^, Xunming Ji^1, 2, 3, 5, *^, Chen Zhou^2, 3, *^

1. Department of Neurology, Xuanwu Hospital, Capital Medical University, Beijing, 100053, China.

2. Beijing Institute of Brain Disorders, Laboratory of Brain Disorders, Ministry of Science and Technology, Collaborative Innovation Center for Brain Disorders, Beijing Advanced Innovation Center for Big Data-based Precision Medicine, Capital Medical University, Beijing, 100069, China.

3. Neuro Cardio Vascular Diseases Center, Xuanwu Hospital, Capital Medical University, Beijing, 100053, China.

4. Department of Radiology, China-Japan Friendship Hospital, Beijing, 100029, China.

5. Neuroscience Center, Beijing Shijitan Hospital, Capital Medical University, Beijing, 100038, China.

6. Beijing Advanced Innovation Center for Big Data-Based Precision Medicine, School of Biological Science and Medical Engineering, Beihang University, Beijing, 100191, China.

7. Department of Radiology, Beijing Tiantan Hospital, Capital Medical University, Beijing, 100070, China.

*Corresponding authors:

Chen Zhou

E-mail: [chenzhou2013abc@163.com](mailto:chenzhou2013abc@163.com)

Xunming Ji

E-mail: [jixm@ccmu.edu.cn](mailto:jixm@ccmu.edu.cn)

Jian Dong

E-mail: [dongjianradiology@163.com](mailto:dongjianradiology@163.com)

# These authors have contributed equally to this work and share the first authorship

# Supplementary Materials

## Content

Table S1. Comparison of the caliber of jugular foramen (Right vs. Left) …………….….…3

Table S2. Comparison of the caliber of jugular foramen (female vs. male) …………..……4

Table S3. Comparison of bilateral jugular foramen calibre narrowing rates (Right-type vs. Left-type) ………………………………………………………………..……………...………………5

Table S4. Comparison of bilateral jugular foramen calibre narrowing rates (females vs males) …………………………………………………………….…………………….…….…...6

Table S5. Demographic data, symptomatic data, and imaging features of CVOI with traditional classification..……………………………………………………………..……………………….7

Table S6. Demographic data, symptomatic data, and imaging features of CV type CVOI...9

Table S7. Demographic data, symptomatic data, and imaging features of JV type CVOI.…….10

Table S8. Demographic data, symptomatic data, and imaging features of CJV type CVOI.………12

Table S9. Demographic and symptomatic data of newly proposed CVOI classification ….……...14

Figure S1. Determination of optimal thresholds of narrowing rate in bilateral jugular foramen calibre and their predictive performance for imaging-based classification…………………………..…….15

Figure S2. Standardized diagnostic workflow for patients with CVOI.….………………………...17

Table S1. Comparison of the caliber of jugular foramen (Right vs. Left).

| Type | Caliber of jugular foramen (mm) | | *P*-value |
| --- | --- | --- | --- |
|  | Mean (SD) | Range |  |
| Balanced type (n = 115) |  |  | 0.847 |
| Right | 8.14 ± 1.04 | 4.59 - 10.52 |  |
| Left | 8.12 ± 0.99 | 5.15 - 10.97 |  |
| Dominant type (n = 66) |  |  |  |
| Right dominant type (n = 49) |  |  | 0.000** |
| Right | 9.27 ± 1.32 | 6.86 - 13.3 |  |
| Left | 6.53 ± 0.90 | 4.92 - 9.11 |  |
| Left dominant type (n = 17) |  |  | 0.000** |
| Right | 6.34 ± 1.29 | 4.08 - 8.17 |  |
| Left | 8.99 ± 1.84 | 6.17 - 12.40 |  |
| Dysplastic type (n = 90) |  |  |  |
| Right dysplastic type (n = 21) |  |  | 0.000** |
| Right | 5.04 ± 1.07 | 2.85 - 7.00 |  |
| Left | 10.18 ± 1.54 | 7.23 - 12.60 |  |
| Left dysplastic type (n = 69) |  |  | 0.000** |
| Right | 9.89 ± 1.63 | 6.70 - 16.00 |  |
| Left | 4.44 ± 1.12 | 2.18 - 7.00 |  |

Note: ** indicates statistical significance as *P*-value < 0.01. SD, standard deviation.

Table S2. Comparison of the caliber of jugular foramen (female vs. male).

| Type | Caliber of jugular foramen (mm), Mean (SD) | | *P*-value |
| --- | --- | --- | --- |
|  | Male | Female |  |
| Balanced type (n = 115) |  |  |  |
| Right | 8.14 ± 1.09 | 8.13 ± 0.98 | 0.753 |
| Left | 8.24 ± 1.03 | 8.00 ± 0.94 | 0.193 |
| Dominant type (n = 66) |  |  |  |
| Right dominant type (n = 49) |  |  |  |
| Right | 9.96 ± 1.67 | 8.90 ± 1.06 | 0.019* |
| Left | 6.86 ± 0.99 | 6.40 ± 0.84 | 0.100 |
| Left dominant type (n = 17) |  |  |  |
| Right | 6.85 ± 1.37 | 6.19 ± 1.29 | 0.385 |
| Left | 9.60 ± 1.81 | 8.80 ± 1.88 | 0.463 |
| Dysplastic type (n = 90) |  |  |  |
| Right dysplastic type (n = 21) |  |  |  |
| Right | 5.02 ± 1.10 | 5.07 ± 1.09 | 0.925 |
| Left | 10.22 ± 1.50 | 10.12 ± 1.69 | 0.886 |
| Left dysplastic type (n = 69) |  |  |  |
| Right | 9.81 ± 1.55 | 9.97 ± 1.72 | 0.710 |
| Left | 4.60 ± 1.13 | 4.28 ± 1.11 | 0.247 |

Note: * indicates statistical significance as *P*-value < 0.05. SD, standard deviation.

Table S3. Comparison of bilateral jugular foramen calibre narrowing rates (Right-type vs. Left-type).

| Type | Degree of narrowing | | *P*-value |
| --- | --- | --- | --- |
|  | Mean (SD) | Range |  |
| Balanced type (n = 115) | 0.0523 ± 0.0551 | 0.00 - 0.19 | - |
| Dominant type (n = 66) | 0.2939 ± 0.0489 | 0.20 - 0.39 | 0.889 |
| Right dominant type (n = 49) | 0.2944 ± 0.0452 | 0.21 - 0.39 |  |
| Left dominant type (n = 17) | 0.2924 ± 0.0599 | 0.20 - 0.37 |  |
| Dysplastic type (n = 90) | 0.5389 ± 0.0943 | 0.40 - 0.75 | 0.093 |
| Right dysplastic type (n = 21) | 0.5057 ± 0.0686 | 0.42 - 0.69 |  |
| Left dysplastic type (n = 69) | 0.5490 ± 0.0992 | 0.40 - 0.75 |  |

Note: SD, standard deviation.

Table S4. Comparison of bilateral jugular foramen calibre narrowing rates (females vs males).

| Type | Degree of narrowing, Mean (SD) | | *P*-value |
| --- | --- | --- | --- |
|  | Male | Female |  |
| Balanced type (n = 115) | 0.0550 ± 0.0553 | 0.0493 ± 0.0553 | 0.468 |
| Dominant type (n = 66) | 0.3028 ± 0.0520 | 0.2906 ± 0.0478 | 0.369 |
| Right dominant type (n = 49) | 0.3077 ± 0.0453 | 0.2891 ± 0.0447 | 0.198 |
| Left dominant type (n = 17) | 0.2859 ± 0.0772 | 0.2944 ± 0.0573 | 0.812 |
| Dysplastic type (n = 90) | 0.5255 ± 0.0871 | 0.5530 ± 0.1005 | 0.209 |
| Right dysplastic type (n = 21) | 0.5132 ± 0.0568 | 0.4958 ± 0.0844 | 0.227 |
| Left dysplastic type (n = 69) | 0.5299 ± 0.0959 | 0.5677 ± 0.1001 | 0.114 |

Note: SD, standard deviation.

Table S5. Demographic data, symptomatic data, and imaging features of CVOI with traditional classification.

|  | All CVOI (n = 245) | Balanced type (n = 99) | Dominant type (n = 56) | Dysplastic type (n = 90) | *P*-value |
| --- | --- | --- | --- | --- | --- |
| Demographics | |  |  |  |  |
| Age (years, mean ± SD) | 53.34 ± 13.37 | 53.17 ± 13.48 | 55.13 ± 12.96 | 52.42 ± 13.53 | 0.489 |
| Gender (female) | 127 (51.8%) | 44 (44.4%) ^b^ | 39 (69.6%) ^a, c^ | 44 (48.9%) ^b^ | 0.008** |
| Clinical symptoms | | | | | |
| Dizziness | 136 (55.5%) | 54 (54.5%) | 36 (64.3%) | 46 (51.1%) | 0.288 |
| Headache | 104 (42.4%) | 41 (41.4%) | 26 (46.4%) | 37 (41.1%) | 0.790 |
| Visual impairment | 39 (15.9%) | 12 (12.1%) | 10 (17.9%) | 17 (18.9%) | 0.403 |
| Tinnitus | 157 (64.1%) | 63 (63.6%) | 38 (67.9%) | 56 (62.2%) | 0.782 |
| Tinnitus cerebri | 213 (86.9%) | 84 (84.8%) | 47 (83.9%) | 82 (91.1%) | 0.332 |
| Subjective hearing loss | 82 (33.5%) | 34 (34.3%) | 22 (39.3%) | 26 (28.9%) | 0.420 |
| Emotion abnormality | 86 (35.1%) | 36 (36.4%) | 20 (35.7%) | 30 (33.3%) | 0.904 |
| Memory loss | 65 (26.5%) | 27 (27.3%) | 16 (28.6%) | 22 (24.4%) | 0.840 |
| Sleep disorder | 127 (51.8%) | 54 (54.5%) | 29 (51.8%) | 44 (48.9%) | 0.739 |
| Neck discomfort | 167 (68.2%) | 64 (64.6%) ^b^ | 47 (83.9%) ^a, c^ | 56 (62.2%) ^b^ | 0.015* |
| Past history | |  |  |  |  |
| Cerebrovascular disease^#^ | 103 (42.0%) | 46 (46.5%) | 30 (53.6%) ^c^ | 27 (30.0%) ^b^ | 0.01* |
| Hypertension | 86 (35.1%) | 37 (37.4%) | 20 (35.7%) | 29 (32.2%) | 0.755 |
| Diabetes mellitus | 38 (15.5%) | 11 (11.1%) | 11 (19.6%) | 16 (17.8%) | 0.280 |
| Dyslipidemia | 88 (35.9%) | 38 (38.4%) | 19 (33.9%) | 31 (34.4%) | 0.801 |
| Cardiovascular events^&^ | 41 (16.7%) | 20 (20.2%) | 10 (17.9%) | 11 (12.2%) | 0.330 |
| CVSS etiology | |  |  |  |  |
| LTS | 80 (32.7%) | 26 (26.3%) | 24 (42.9%) | 30 (33.3%) | 0.105 |
| AGs | 78 (31.8%) | 26 (26.3%) | 23 (41.1%) | 29 (32.2%) | 0.163 |
| Others | 2 (0.8%) | 0 (0.0%) | 1 (1.8%) | 1 (1.1%) | 0.516 |
| RTS | 72 (29.4%) | 27 (27.3%) | 21 (37.5%) | 24 (26.7%) | 0.315 |
| AGs | 67 (27.3%) | 27 (27.3%) | 17 (30.4%) | 23 (25.6%) | 0.818 |
| Others | 5 (2.0%) | 0 (0.0%) ^b^ | 4 (7.1%) ^a^ | 1 (1.1%) | 0.005** |
| LSigS | 17 (6.9%) | 6 (6.1%) | 4 (7.1%) | 7 (7.8%) | 0.901 |
| AGs | 16 (6.5%) | 6 (6.1%) | 4 (7.1%) | 6 (6.7%) | 0.947 |
| Others | 1 (0.4%) | 0 (0.0%) | 0 (0.0%) | 1 (1.1%) | 0.596 |
| RSigS | 13 (5.3%) | 6 (6.1%) | 2 (3.6%) | 5 (5.6%) | 0.875 |
| AGs | 11 (4.5%) | 6 (6.1%) | 1 (1.8%) | 4 (4.4%) | 0.536 |
| Others | 2 (0.8%) | 0 (0.0%) | 1 (1.8%) | 1 (1.1%) | 0.516 |
| SSS | 12 (4.9%) | 4 (4.0%) | 5 (8.9%) | 3 (3.3%) | 0.328 |
| AGs | 10 (4.1%) | 3 (3.0%) | 4 (7.1%) | 3 (3.3%) | 0.42 |
| Others | 2 (0.8%) | 1 (1.0%) | 1 (1.8%) | 0 (0.0%) | 0.702 |
| SS | 8 (3.3%) | 1 (1.0%) | 3 (5.4%) | 4 (4.4%) | 0.221 |
| AGs | 8 (3.3%) | 1 (1.0%) | 3 (5.4%) | 4 (4.4%) | 0.221 |
| Others | 0 (0.0%) | 0 (0.0%) | 0 (0.0%) | 0 (0.0%) | - |
| IJVS etiology | |  |  |  |  |
| Left | 139 (56.7%) | 66 (66.7%) ^c^ | 36 (64.3%) ^c^ | 37 (41.1%) ^a, b^ | 0.001** |
| LIJV-J1 segment | 12 (4.9%) | 4 (4.0%) | 5 (8.9%) | 3 (3.3%) | 0.328 |
| Osseous compression | 12 (4.9%) | 4 (4.0%) | 5 (8.9%) | 3 (3.3%) | 0.328 |
| Soft-tissue compression | 0 (0.0%) | 0 (0.0%) | 0 (0.0%) | 0 (0.0%) | - |
| LIJV-J2 segment | 12 (4.9%) | 7 (7.1%) | 1 (1.8%) | 4 (4.4%) | 0.381 |
| Osseous compression | 0 (0.0%) | 0 (0.0%) | 0 (0.0%) | 0 (0.0%) | - |
| Soft-tissue compression | 12 (4.9%) | 7 (7.1%) | 1 (1.8%) | 4 (4.4%) | 0.381 |
| LIJV-J3 segment | 129 (52.7%) | 62 (62.6%) ^c^ | 33 (58.9%) ^c^ | 34 (37.8%) ^a, b^ | 0.002** |
| Osseous compression | 125 (51.0%) | 60 (60.6%) ^c^ | 32 (57.1%) ^c^ | 33 (36.7%) ^a, b^ | 0.003** |
| Soft-tissue compression | 4 (1.6%) | 2 (2.0%) | 1 (1.8%) | 1 (1.1%) | 1 |
| Right | 131 (53.5%) | 72 (72.7%) ^b, c^ | 25 (44.6%) ^a^ | 34 (37.8%) ^a^ | 0.000** |
| RIJV-J1 segment | 0 (0.0%) | 0 (0.0%) | 0 (0.0%) | 0 (0.0%) | - |
| Osseous compression | 0 (0.0%) | 0 (0.0%) | 0 (0.0%) | 0 (0.0%) | - |
| Soft-tissue compression | 0 (0.0%) | 0 (0.0%) | 0 (0.0%) | 0 (0.0%) | - |
| RIJV-J2 segment | 9 (3.7%) | 5 (5.1%) | 2 (3.6%) | 2 (2.2%) | 0.627 |
| Osseous compression | 0 (0.0%) | 0 (0.0%) | 0 (0.0%) | 0 (0.0%) | - |
| Soft-tissue compression | 9 (3.7%) | 5 (5.1%) | 2 (3.6%) | 2 (2.2%) | 0.627 |
| RIJV-J3 segment | 129 (52.7%) | 70 (70.7%) ^b, c^ | 24 (42.9%) ^a^ | 35 (38.9%) ^a^ | 0.000** |
| Osseous compression | 125 (51.0%) | 70 (70.7%) ^b, c^ | 21 (37.5%) ^a^ | 34 (37.8%) ^a^ | 0.000** |
| Soft-tissue compression | 4 (1.6%) | 0 (0.0%) | 3 (5.4%) | 1 (1.1%) | 0.02* |

Note: All numbers are n (%) unless stated otherwise. * indicates statistical significance as *P*-value < 0.05; ** indicates statistical significance as *P*-value < 0.01. ^a^ indicates that there is a significant difference between this group and balanced type (*P* < 0.05); ^b^ indicates that there is a significant difference between this group and dominant type (*P* < 0.05); ^c^ indicates that there is a significant difference between this group and dysplastic type (*P* < 0.05); ^#^Cerebrovascular diseases refer to cerebral artery stenosis and ischaemic stroke; ^&^Cardiovascular events refer to diseases related to the cardiovascular system, such as myocardial infarction, heart failure, and arrhythmia. CVSS, cerebral venous sinus stenosis; IJVS, internal jugular vein stenosis; SSS, superior sagittal sinus; TS, transverse sinus; LTS, left transverse sinus; RTS, right transverse sinus; SigS, sigmoid sinus; LSigS, left sigmoid sinus; RSigS, right sigmoid sinus; SS, straight sinus; AGs, arachnoid granules; LIJV, left internal jugular vein; RIJV, right internal jugular vein; CVOI, cerebral venous outflow insufficiency; SD, standard deviation.

Table S6. Demographic data, symptomatic data, and imaging features of CV type CVOI.

|  | All CV (n = 29) | CV1 (n = 5) | CV2 (n = 10) | CV3 (n = 2) | CV4 (n = 8) | CV5 (n = 4) | *P*-value |
| --- | --- | --- | --- | --- | --- | --- | --- |
| Demographics | |  |  |  |  |  |  |
| Age (years, mean ± SD) | 53.31 ± 11.52 | 56.40 ± 12.40 | 47.90 ± 10.20 | 50.50 ± 24.75 | 57.38 ± 9.58 | 56.25 ± 11.32 | 0.435 |
| Gender (female) | 21 (72.4%) | 3 (60.0%) | 9 (90.0%) | 1 (50.0%) | 6 (75.0%) | 2 (50.0%) | 0.400 |
| Clinical symptoms | |  |  |  |  |  |  |
| Dizziness | 17 (58.6%) | 2 (40.0%) | 3 (30.0%) ^d^ | 2 (100.0%) | 8 (100.0%) ^b^ | 2 (50.0%) | 0.010* |
| Headache | 13 (44.8%) | 4 (80.0%) | 5 (50.0%) | 0 (0.0%) | 4 (50.0%) | 0 (0.0%) | 0.126 |
| Visual impairment | 2 (6.9%) | 0 (0.0%) | 0 (0.0%) | 0 (0.0%) | 1 (12.5%) | 1 (25.0%) | 0.372 |
| Tinnitus | 16 (55.2%) | 2 (40.0%) | 6 (60.0%) | 1 (50.0%) | 5 (62.5%) | 2 (50.0%) | 0.949 |
| Tinnitus cerebri | 23 (79.3%) | 4 (80.0%) | 8 (80%) | 2 (100.0%) | 6 (75.0%) | 3 (75.0%) | 1.000 |
| Subjective hearing loss | 12 (41.4%) | 2 (40.0%) | 4 (40.0%) | 1 (50.0%) | 4 (50.0%) | 1 (25.0%) | 0.973 |
| Emotion abnormality | 10 (34.5%) | 3 (60.0%) | 3 (30.0%) | 0 (0.0%) | 3 (37.5%) | 1 (25.0%) | 0.684 |
| Memory loss | 7 (24.1%) | 2 (40.0%) | 3 (30.0%) | 0 (0.0%) | 1 (12.5%) | 1 (25.0%) | 0.860 |
| Sleep disorder | 15 (51.7%) | 5 (100.0%) | 5 (50.0%) | 0 (0.0%) | 4 (50.0%) | 1 (25.0%) | 0.096 |
| Neck discomfort | 21 (72.4%) | 4 (80.0%) | 5 (50.0%) | 2 (100.0%) | 8 (100.0%) | 2 (50.0%) | 0.090 |
| CVSS etiology | |  |  |  |  |  |  |
| LTS | 21 (72.4%) | 3 (60.0%) | 10 (100.0%) ^e^ | 1 (50.0%) | 6 (75.0%) | 1 (25.0%) ^b^ | 0.023* |
| AGs | 20 (69.0%) | 3 (60.0%) | 10 (100.0%) ^e^ | 1 (50.0%) | 5 (62.5%) | 1 (25.0%) ^b^ | 0.024* |
| Others | 1 (3.4%) | 0 (0.0%) | 0 (0.0%) | 0 (0.0%) | 1 (12.5%) | 0 (0.0%) | 0.655 |
| RTS | 15 (51.7%) | 2 (40.0%) | 10 (100.0%) ^d, e^ | 1 (50.0%) | 1 (12.5%) ^b^ | 1 (25.0%) ^b^ | 0.000** |
| AGs | 14 (48.3%) | 2 (40.0%) | 9 (90.0%) ^d^ | 1 (50.0%) | 1 (12.5%) ^b^ | 1 (25.0%) | 0.006** |
| Others | 1 (3.4%) | 0 (0.0%) | 1 (10.0%) | 0 (0.0%) | 0 (0.0%) | 0 (0.0%) | 1.000 |
| LSigS | 9 (31.0%) | 0 (0.0%) ^b^ | 7 (70.0%) ^a, c, d, e^ | 0 (0.0%) ^b^ | 2 (25.0%) ^b^ | 0 (0.0%) ^b^ | 0.019* |
| AGs | 9 (31.0%) | 0 (0.0%) ^b^ | 7 (70.0%) ^a, c, d, e^ | 0 (0.0%) ^b^ | 2 (25.0%) ^b^ | 0 (0.0%) ^b^ | 0.019* |
| Others | 0 (0.0%) | 0 (0.0%) | 0 (0.0%) | 0 (0.0%) | 0 (0.0%) | 0 (0.0%) | - |
| RSigS | 7 (24.1%) | 0 (0.0%) | 7 (70.0%) ^d^ | 0 (0.0%) | 0 (0.0%) ^b^ | 0 (0.0%) | 0.001** |
| AGs | 7 (24.1%) | 0 (0.0%) | 7 (70.0%) ^d^ | 0 (0.0%) | 0 (0.0%) ^b^ | 0 (0.0%) | 0.001** |
| Others | 0 (0.0%) | 0 (0.0%) | 0 (0.0%) | 0 (0.0%) | 0 (0.0%) | 0 (0.0%) | - |
| SSS | 2 (6.9%) | 0 (0.0%) | 1 (10.0%) | 0 (0.0%) | 1 (12.5%) | 0 (0.0%) | 1.000 |
| AGs | 2 (6.9%) | 0 (0.0%) | 1 (10.0%) | 0 (0.0%) | 1 (12.5%) | 0 (0.0%) | 1.000 |
| Others | 0 (0.0%) | 0 (0.0%) | 0 (0.0%) | 0 (0.0%) | 0 (0.0%) | 0 (0.0%) | - |
| SS | 0 (0.0%) | 0 (0.0%) | 0 (0.0%) | 0 (0.0%) | 0 (0.0%) | 0 (0.0%) | - |
| AGs | 0 (0.0%) | 0 (0.0%) | 0 (0.0%) | 0 (0.0%) | 0 (0.0%) | 0 (0.0%) | - |
| Others | 0 (0.0%) | 0 (0.0%) | 0 (0.0%) | 0 (0.0%) | 0 (0.0%) | 0 (0.0%) | - |

Note: All numbers are n (%) unless stated otherwise. * indicates statistical significance as *P*-value < 0.05; ** indicates statistical significance as *P*-value < 0.01. ^a^ indicates that there is a significant difference between this group and CV1 type (*P* < 0.05); ^b^ indicates that there is a significant difference between this group and CV2 type (*P* < 0.05); ^c^ indicates that there is a significant difference between this group and CV3 type (*P* < 0.05); ^d^ indicates that there is a significant difference between this group and CV4 type (*P* < 0.05); ^e^ indicates that there is a significant difference between this group and CV5 type (*P* < 0.05); CVSS, cerebral venous sinus stenosis; SSS, superior sagittal sinus; TS, transverse sinus; LTS, left transverse sinus; RTS, right transverse sinus; SigS, sigmoid sinus; LSigS, left sigmoid sinus; RSigS, right sigmoid sinus; SS, straight sinus; AGs, arachnoid granules, CVOI, cerebral venous outflow insufficiency; CV, intracranial type of CVOI; SD, standard deviation.

Table S7. Demographic data, symptomatic data, and imaging features of JV type CVOI.

|  | All JV (n = 77) | JV1 (n = 25) | JV2 (n = 41) | JV3 (n = 1) | JV4 (n = 10) | JV5 (n = 0) | *P*-value |
| --- | --- | --- | --- | --- | --- | --- | --- |
| Demographics | |  |  |  |  |  |  |
| Age (years, mean ± SD) | 52.32 ± 13.68 | 54.60 ± 14.03 | 50.37 ± 13.70 | 51.00 ± 0.00 | 54.80 ± 13.52 | - | 0.843 |
| Gender (female) | 34 (44.2%) | 13 (52.0%) | 14 (34.1%) | 0 (0.0%) | 7 (70.0%) | 0 (0.0%) | 0.091 |
| Clinical symptoms | | |  |  |  |  |  |
| Dizziness | 40 (51.9%) | 15 (60.0%) | 21 (51.2%) | 0 (0.0%) | 4 (40.0%) | 0 (0.0%) | 0.555 |
| Headache | 32 (41.6%) | 11 (44.0%) | 15 (36.6%) | 0 (0.0%) | 6 (60.0%) | 0 (0.0%) | 0.444 |
| Visual impairment | 7 (9.1%) | 4 (16.0%) | 3 (7.3%) | 0 (0.0%) | 0 (0.0%) | 0 (0.0%) | 0.444 |
| Tinnitus | 54 (70.1%) | 18 (72.0%) | 27 (65.9%) | 1 (100.0%) | 8 (80.0%) | 0 (0.0%) | 0.816 |
| Tinnitus cerebri | 68 (88.3%) | 21 (84.0%) | 39 (95.1%) | 1 (100.0%) | 7 (70.0%) | 0 (0.0%) | 0.091 |
| Subjective hearing loss | 28 (36.4%) | 10 (40.0%) | 15 (36.6%) | 0 (0.0%) | 3 (30.0%) | 0 (0.0%) | 0.933 |
| Emotion abnormality | 32 (41.6%) | 12 (48.0%) | 16 (39.0%) | 0 (0.0%) | 4 (40.0%) | 0 (0.0%) | 0.887 |
| Memory loss | 24 (31.2%) | 9 (36.0%) | 11 (26.8%) | 0 (0.0%) | 4 (40.0%) | 0 (0.0%) | 0.734 |
| Sleep disorder | 41 (53.2%) | 13 (52.0%) | 24 (58.5%) | 0 (0.0%) | 4 (40.0%) | 0 (0.0%) | 0.500 |
| Neck discomfort | 54 (70.1%) | 18 (72.0%) | 26 (63.4%) | 1 (100.0%) | 9 (90.0%) | 0 (0.0%) | 0.388 |
| IJVS etiology | |  |  |  |  |  |  |
| Left | 60 (77.9%) | 10 (40.0%) ^b, d^ | 41 (100.0%) ^a, c^ | 0 (0.0%) ^b^ | 9 (90.0%) ^a^ | 0 (0.0%) | 0.000** |
| LIJV-J1 segment | 4 (5.2%) | 1 (4.0%) | 2 (4.9%) | 0 (0.0%) | 1 (10.0%) | 0 (0.0%) | 0.621 |
| Osseous compression | 4 (5.2%) | 1 (4.0%) | 2 (4.9%) | 0 (0.0%) | 1 (10.0%) | 0 (0.0%) | 0.362 |
| Soft-tissue compression | 0 (0.0%) | 0 (0.0%) | 0 (0.0%) | 0 (0.0%) | 0 (0.0%) | 0 (0.0%) | - |
| LIJV-J2 segment | 4 (5.2%) | 2 (8.0%) | 2 (4.9%) | 0 (0.0%) | 0 (0.0%) | 0 (0.0%) | 0.803 |
| Osseous compression | 0 (0.0%) | 0 (0.0%) | 0 (0.0%) | 0 (0.0%) | 0 (0.0%) | 0 (0.0%) | - |
| Soft-tissue compression | 4 (5.2%) | 2 (8.0%) | 2 (4.9%) | 0 (0.0%) | 0 (0.0%) | 0 (0.0%) | 0.803 |
| LIJV-J3 segment | 57 (74.0%) | 9 (36.0%) ^b, d^ | 39 (95.1%) ^a, c^ | 0 (0.0%) ^b^ | 9 (90.0%) ^a^ | 0 (0.0%) | 0.000** |
| Osseous compression | 55 (71.4%) | 9 (36.0%) ^b, d^ | 37 (90.2%) ^a, c^ | 0 (0.0%) ^b^ | 9 (90.0%) ^a^ | 0 (0.0%) | 0.000** |
| Soft-tissue compression | 2 (2.6%) | 0 (0.0%) | 2 (4.9%) | 0 (0.0%) | 0 (0.0%) | 0 (0.0%) | 0.650 |
| Right | 58 (75.3%) | 15 (60.0%) ^b, d^ | 41 (100.0%) ^a, d^ | 1 (100.0%) | 1 (10.0%) ^a, b^ | 0 (0.0%) | 0.000** |
| RIJV-J1 segment | 0 (0.0%) | 0 (0.0%) | 0 (0.0%) | 0 (0.0%) | 0 (0.0%) | 0 (0.0%) | - |
| Osseous compression | 0 (0.0%) | 0 (0.0%) | 0 (0.0%) | 0 (0.0%) | 0 (0.0%) | 0 (0.0%) | - |
| Soft-tissue compression | 0 (0.0%) | 0 (0.0%) | 0 (0.0%) | 0 (0.0%) | 0 (0.0%) | 0 (0.0%) | - |
| RIJV-J2 segment | 4 (5.2%) | 0 (0.0%) | 4 (9.8%) | 0 (0.0%) | 0 (0.0%) | 0 (0.0%) | 0.300 |
| Osseous compression | 0 (0.0%) | 0 (0.0%) | 0 (0.0%) | 0 (0.0%) | 0 (0.0%) | 0 (0.0%) | - |
| Soft-tissue compression | 4 (5.2%) | 0 (0.0%) | 4 (9.8%) | 0 (0.0%) | 0 (0.0%) | 0 (0.0%) | 0.300 |
| RIJV-J3 segment | 57 (74.0%) | 15 (60.0%) ^b, d^ | 40 (97.6%) ^a, d^ | 1 (100.0%) | 1 (10.0%) ^a, b^ | 0 (0.0%) | 0.000** |
| Osseous compression | 55 (71.4%) | 15 (60.0%) ^b, d^ | 38 (92.7%) ^a, d^ | 1 (100.0%) | 1 (10.0%) ^a, b^ | 0 (0.0%) | 0.000** |
| Soft-tissue compression | 2 (2.6%) | 0 (0.0%) | 2 (4.9%) | 0 (0.0%) | 0 (0.0%) | 0 (0.0%) | 0.650 |

Note: All numbers are n (%) unless stated otherwise. ** indicates statistical significance as *P*-value < 0.01. ^a^ indicates that there is a significant difference between this group and JV1 type (*P* < 0.05); ^b^ indicates that there is a significant difference between this group and JV2 type (*P* < 0.05); ^c^ indicates that there is a significant difference between this group and JV3 type (*P* < 0.05); ^d^ indicates that there is a significant difference between this group and JV4 type (*P* < 0.05); IJVS, internal jugular vein stenosis; LIJV, left internal jugular vein; RIJV, right internal jugular vein; CVOI, cerebral venous outflow insufficiency; JV, extracranial type of CVOI; SD, standard deviation.

Table S8. Demographic data, symptomatic data, and imaging features of CJV type CVOI.

|  | All CJV (n = 139) | CJV1 (n = 5) | CJV2 (n = 89) | CJV3 (n = 1) | CJV4 (n = 6) | CJV5 (n = 38) | *P*-value |
| --- | --- | --- | --- | --- | --- | --- | --- |
| Demographics | |  |  |  |  |  |  |
| Age (years, mean ± SD) | 53.91 ± 13.60 | 52.60 ± 20.53 | 55.10 ± 14.00 | 64.00 ± 0.00 | 60.00 ± 7.54 | 50.08 ± 11.86 | 0.206 |
| Gender (female) | 72 (51.8%) | 3 (60.0%) | 46 (52.8%) | 1 (100.0%) | 4 (66.7%) | 17 (44.7%) | 0.715 |
| Clinical symptoms | |  |  |  |  |  |  |
| Dizziness | 79 (56.8%) | 2 (40.0%) | 53 (59.6%) | 1 (100.0%) | 5 (83.3%) | 18 (47.4%) | 0.311 |
| Headache | 59 (42.4%) | 1 (20.0%) | 37 (41.6%) | 1 (100.0%) | 2 (33.3%) | 18 (47.4%) | 0.637 |
| Visual impairment | 30 (21.6%) | 1 (20.0%) | 17 (19.1%) | 0 (0.0%) | 4 (66.7%) | 8 (21.1%) | 0.104 |
| Tinnitus | 87 (62.6%) | 4 (80.0%) | 55 (61.8%) | 1 (100.0%) | 3 (50.0%) | 24 (63.2%) | 0.907 |
| Tinnitus cerebri | 122 (87.8%) | 5 (100.0%) | 76 (85.4%) | 1 (100.0%) | 6 (100.0%) | 34 (89.5%) | 0.878 |
| Subjective hearing loss | 42 (30.2%) | 2 (40.0%) | 26 (29.2%) | 0 (0.0%) | 3 (50.0%) | 11 (28.9%) | 0.766 |
| Emotion abnormality | 44 (31.7%) | 2 (40.0%) | 29 (32.6%) | 0 (0.0%) | 1 (16.7%) | 12 (31.6%) | 0.928 |
| Memory loss | 34 (24.5%) | 3 (60.0%) | 19 (21.3%) | 1 (100.0%) | 1 (16.7%) | 10 (26.3%) | 0.124 |
| Sleep disorder | 71 (51.1%) | 3 (60.0%) | 48 (53.9%) | 1 (100.0%) | 3 (50.0%) | 16 (42.1%) | 0.633 |
| Neck discomfort | 92 (66.2%) | 3 (60.0%) | 57 (64.0%) | 1 (100.0%) | 6 (100.0%) | 25 (65.8%) | 0.436 |
| CVSS etiology | |  |  |  |  |  |  |
| LTS | 59 (42.4%) | 2 (40.0%) | 48 (53.9%) ^e^ | 0 (0.0%) | 2 (33.3%) | 7 (18.4%) ^b^ | 0.001** |
| AGs | 58 (41.7%) | 2 (40.0% | 47 (52.8%) ^e^ | 0 (0.0%) | 2 (33.3%) | 7 (18.4%) ^b^ | 0.002** |
| Others | 1 (0.7%) | 0 (0.0%) | 1 (1.1%) | 0 (0.0%) | 0 (0.0%) | 0 (0.0%) | 1.000 |
| RTS | 57 (41.0%) | 3 (60.0%) ^e^ | 50 (56.2%) ^e^ | 1 (100.0%) ^e^ | 2 (33.3%) | 1 (2.6%) ^a, b, c^ | 0.000** |
| AGs | 53 (38.1%) | 3 (60.0%) ^e^ | 47 (52.8%) ^e^ | 1 (100.0%) ^e^ | 1 (16.7%) | 1 (2.6%) ^a, b, c^ | 0.000** |
| Others | 4 (2.9%) | 0 (0.0%) | 3 (3.4%) | 0 (0.0%) | 1 (16.7%) | 0 (0.0%) | 0.201 |
| LSigS | 8 (5.8%) | 0 (0.0%) | 6 (6.7%) | 0 (0.0%) | 1 (16.7%) | 1 (2.6%) | 0.459 |
| AGs | 7 (5.0%) | 0 (0.0%) | 5 (5.6%) | 0 (0.0%) | 1 (16.7%) | 1 (2.6%) | 0.518 |
| Others | 1 (0.7%) | 0 (0.0%) | 1 (1.1%) | 0 (0.0%) | 0 (0.0%) | 0 (0.0%) | 1.000 |
| RSigS | 6 (4.3%) | 0 (0.0%) | 6 (6.7%) | 0 (0.0%) | 0 (0.0%) | 0 (0.0%) | 0.527 |
| AGs | 4 (2.9%) | 0 (0.0%) | 4 (4.5%) | 0 (0.0%) | 0 (0.0%) | 0 (0.0%) | 0.525 |
| Others | 2 (1.4%) | 0 (0.0%) | 2 (2.2%) | 0 (0.0%) | 0 (0.0%) | 0 (0.0%) | 1.000 |
| SSS | 10 (7.2%) | 0 (0.0%) | 8 (9.0%) | 0 (0.0%) | 2 (33.3%) ^e^ | 0 (0.0%) ^d^ | 0.039* |
| AGs | 8 (5.8%) | 0 (0.0%) | 6 (6.7%) | 0 (0.0%) | 2 (33.3%) | 0 (0.0%) | 0.054 |
| Others | 2 (1.4%) | 0 (0.0%) | 2 (2.2%) | 0 (0.0%) | 0 (0.0%) | 0 (0.0%) | 1.000 |
| SS | 8 (5.8%) | 0 (0.0%) | 6 (6.7%) | 0 (0.0%) | 0 (0.0%) | 2 (5.3%) | 1.000 |
| AGs | 8 (5.8%) | 0 (0.0%) | 6 (6.7%) | 0 (0.0%) | 0 (0.0%) | 2 (5.3%) | 1.000 |
| Others | 0 (0.0%) | 0 (0.0%) | 0 (0.0%) | 0 (0.0%) | 0 (0.0%) | 0 (0.0%) | - |
| IJVS etiology | |  |  |  |  |  |  |
| Left | 79 (56.8%) | 2 (40.0%) | 63 (70.8%) ^e^ | 0 (0.0%) | 3 (50.0%) | 11 (28.9%) ^b^ | 0.000** |
| LIJV-J1 segment | 8 (5.8%) | 0 (0.0%) | 8 (9.0%) | 0 (0.0%) | 0 (0.0%) | 0 (0.0%) | 0.293 |
| Osseous compression | 8 (5.8%) | 0 (0.0%) | 8 (9.0%) | 0 (0.0%) | 0 (0.0%) | 0 (0.0%) | 0.293 |
| Soft-tissue compression | 0 (0.0%) | 0 (0.0%) | 0 (0.0%) | 0 (0.0%) | 0 (0.0%) | 0 (0.0%) | - |
| LIJV-J2 segment | 8 (5.8%) | 0 (0.0%) | 8 (9.0%) | 0 (0.0%) | 0 (0.0%) | 0 (0.0%) | 0.293 |
| Osseous compression | 0 (0.0%) | 0 (0.0%) | 0 (0.0%) | 0 (0.0%) | 0 (0.0%) | 0 (0.0%) |  |
| Soft-tissue compression | 8 (5.8%) | 0 (0.0%) | 8 (9.0%) | 0 (0.0%) | 0 (0.0%) | 0 (0.0%) | 0.293 |
| LIJV-J3 segment | 72 (51.8%) | 2 (40.0%) | 56 (62.9%) ^e^ | 0 (0.0%) | 3 (50.0%) | 11 (28.9%) ^b^ | 0.002** |
| Osseous compression | 70 (50.4%) | 2 (40.0%) | 54 (60.7%) ^e^ | 0 (0.0%) | 3 (50.0%) | 11 (28.9%) ^b^ | 0.006** |
| Soft-tissue compression | 2 (1.4%) | 0 (0.0%) | 2 (2.2%) | 0 (0.0%) | 0 (0.0%) | 0 (0.0%) | 1.000 |
| Right | 73 (52.5%) | 3 (60.0%) ^e^ | 62 (69.7%) ^e^ | 1 (100.0%) | 3 (50.0%) | 4 (10.5%) ^a, b^ | 0.000** |
| RIJV-J1 segment | 0 (0.0%) | 0 (0.0%) | 0 (0.0%) | 0 (0.0%) | 0 (0.0%) | 0 (0.0%) | - |
| Osseous compression | 0 (0.0%) | 0 (0.0%) | 0 (0.0%) | 0 (0.0%) | 0 (0.0%) | 0 (0.0%) | - |
| Soft-tissue compression | 0 (0.0%) | 0 (0.0%) | 0 (0.0%) | 0 (0.0%) | 0 (0.0%) | 0 (0.0%) | - |
| RIJV-J2 segment | 5 (3.6%) | 0 (0.0%) | 4 (4.5%) | 0 (0.0%) | 0 (0.0%) | 1 (2.6%) | 1.000 |
| Osseous compression | 0 (0.0%) | 0 (0.0%) | 0 (0.0%) | 0 (0.0%) | 0 (0.0%) | 0 (0.0%) | - |
| Soft-tissue compression | 5 (3.6%) | 0 (0.0%) | 4 (4.5%) | 0 (0.0%) | 0 (0.0%) | 1 (2.6%) | 1.000 |
| RIJV-J3 segment | 72 (51.8%) | 3 (60.0%) ^e^ | 61 (68.5%) ^e^ | 1 (100.0%) | 3 (50.0%) | 4 (10.5%) ^a, b^ | 0.000** |
| Osseous compression | 70 (50.4%) | 3 (60.0%) ^e^ | 59 (66.3%) ^e^ | 1 (100.0%) | 3 (50.0%) | 4 (10.5%) ^a, b^ | 0.000** |
| Soft-tissue compression | 2 (1.4%) | 0 (0.0%) | 2 (2.2%) | 0 (0.0%) | 0 (0.0%) | 0 (0.0%) | 1.000 |

Note: All numbers are n (%) unless stated otherwise. * indicates statistical significance as *P*-value < 0.05; ** indicates statistical significance as p-value < 0.01. ^a^ indicates that there is a significant difference between this group and CJV1 type (*P* < 0.05); ^b^ indicates that there is a significant difference between this group and CJV2 type (*P* < 0.05); ^c^ indicates that there is a significant difference between this group and CJV3 type (*P* < 0.05); ^d^ indicates that there is a significant difference between this group and CJV4 type (*P* < 0.05); ^e^ indicates that there is a significant difference between this group and CJV5 type (*P* < 0.05). CVSS, cerebral venous sinus stenosis; IJVS, internal jugular vein stenosis; SSS, superior sagittal sinus; TS, transverse sinus; LTS, left transverse sinus; RTS, right transverse sinus; SigS, sigmoid sinus; LSigS, left sigmoid sinus; RSigS, right sigmoid sinus; SS, straight sinus; AGs, arachnoid granules; LIJV, left internal jugular vein; RIJV, right internal jugular vein; CVOI, cerebral venous outflow insufficiency; CJV, intracranial and extracranial tandem type of CVOI; SD, standard deviation.

Table S9. Demographic and symptomatic data of newly proposed CVOI classification.

|  | All CVOI (n = 245) | CV (n = 29) | JV (n = 77) | CJV (n = 139) | *P*-value |
| --- | --- | --- | --- | --- | --- |
| Demographics | |  |  |  |  |
| Age (years, mean ± SD) | 53.34 ± 13.37 | 53.31 ± 11.52 | 52.32 ± 13.68 | 53.91 ± 13.60 | 0.706 |
| Gender (female) | 127 (51.8%) | 21 (72.4%) ^b, c^ | 34 (44.2%) ^a^ | 72 (51.8%) ^a^ | 0.034* |
| Clinical symptoms | |  |  |  |  |
| Dizziness | 136 (55.5%) | 17 (58.6%) | 40 (51.9%) | 79 (56.8%) | 0.738 |
| Headache | 104 (42.4%) | 13 (44.8%) | 32 (41.6%) | 59 (42.4%) | 0.955 |
| Visual impairment | 39 (15.9%) | 2 (6.9%) ^b, c^ | 7 (9.1%) ^a^ | 30 (21.6%) ^a^ | 0.024* |
| Tinnitus | 157 (64.1%) | 16 (55.2%) | 54 (70.1%) | 87 (62.6%) | 0.308 |
| Tinnitus cerebri | 213 (86.9%) | 23 (79.3%) | 68 (88.3%) | 122 (87.8%) | 0.428 |
| Subjective hearing loss | 82 (33.5%) | 12 (41.4%) | 28 (36.4%) | 42 (30.2%) | 0.414 |
| Emotion abnormality | 86 (35.1%) | 10 (34.5%) | 32 (41.6%) | 44 (31.7%) | 0.343 |
| Memory loss | 65 (26.5%) | 7 (24.1%) | 24 (31.2%) | 34 (24.5%) | 0.538 |
| Sleep disorder | 127 (51.8%) | 15 (51.7%) | 41 (53.2%) | 71 (51.1%) | 0.954 |
| Neck discomfort | 167 (68.2%) | 21 (72.4%) | 54 (70.1%) | 92 (66.2%) | 0.730 |
|  |  |  |  |  |  |
| Cerebrovascular disease^#^ | 103 (42.0%) | 12 (41.4%) | 38 (49.4%) | 53 (38.1%) | 0.277 |
| Hypertension | 86 (35.1%) | 11 (37.9%) | 28 (36.4%) | 47 (33.8%) | 0.879 |
| Diabetes mellitus | 38 (15.5%) | 4 (13.8%) | 11 (14.3%) | 23 (16.5%) | 0.905 |
| Dyslipidemia | 88 (35.9%) | 13 (44.8%) | 25 (32.5%) | 50 (36.0%) | 0.497 |
| Cardiovascular events^&^ | 41 (16.7%) | 7 (24.1%) | 12 (15.6%) | 22 (15.8%) | 0.523 |

Note: All numbers are n (%) unless stated otherwise. * indicates statistical significance as *P*-value < 0.05. ^a^ indicates that there is a significant difference between this group and CV type (*P* < 0.05); ^b^ indicates that there is a significant difference between this group and JV type (*P* < 0.05); ^c^ indicates that there is a significant difference between this group and CJV type (*P* < 0.05); ^#^Cerebrovascular diseases refer to cerebral artery stenosis and ischaemic stroke; ^&^Cardiovascular events refer to diseases related to the cardiovascular system, such as myocardial infarction, heart failure, and arrhythmia. CVOI, cerebral venous outflow insufficiency; CV, intracranial type of CVOI; JV, extracranial type of CVOI; CJV, intracranial and extracranial tandem type of CVOI; SD, standard deviation.

Figure S1. Determination of optimal thresholds of narrowing rate in bilateral jugular foramen calibre and their predictive performance for imaging-based classification. (A-B) Histograms showing the distribution of narrowing rates in the bilateral jugular foramen calibre and classification based on optimal cut-off values. The best cut-off points determined by the Youden Index were 0.203 (A) and 0.395 (B), used to stratify subjects into different narrowing rate groups. (C-D) Curves of Youden Index across a range of narrowing rate thresholds. The maximum Youden Index indicated the optimal cut-off values of 0.203 (C) and 0.395 (D), respectively. (E-F) ROC curves evaluating the predictive performance of narrowing rate thresholds (0.203 and 0.395) for imaging-based classification. Both thresholds demonstrated excellent discrimination, with AUC values of 1.000. Abbreviations: ROC, receiver operating characteristic; AUC, area under the curve.


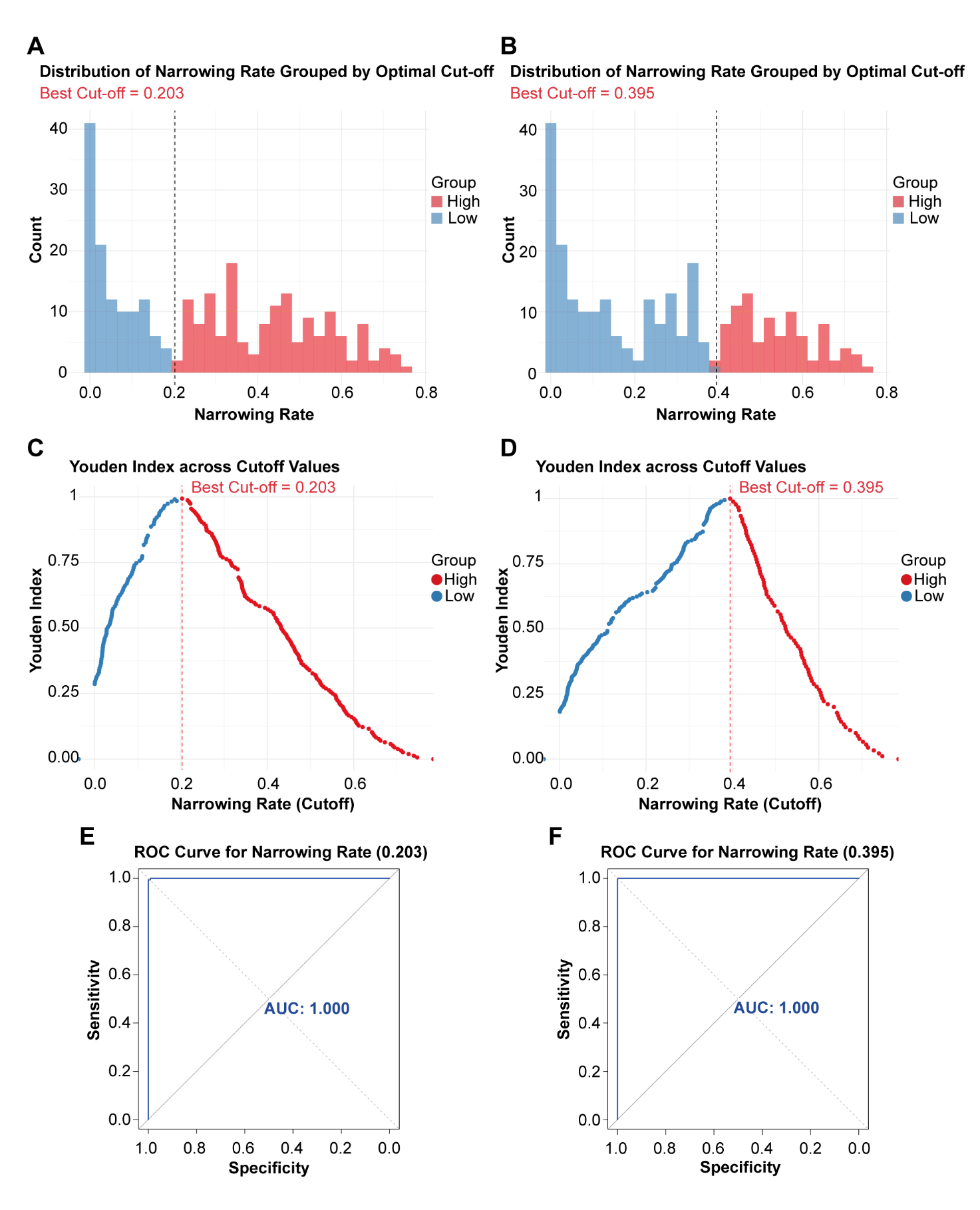


Figure S2. Standardized diagnostic workflow for patients with CVOI. This flowchart outlines the standardized diagnostic approach employed in this study for patients with suspected CVOI-related symptoms. The workflow begins with a multidisciplinary clinical evaluation (including neurology, ophthalmology, otolaryngology, spine surgery, and endocrinology) to exclude alternative systemic etiologies. Subsequent multimodal neuroimaging includes MRA/CTA to rule out intracranial and extracranial arterial pathologies, conventional MRI and CE-MRI to assess structural brain lesions, and BB-MRI to exclude acute or chronic cerebral venous sinus thrombosis. Doppler ultrasound assessments incorporate TCD, CADU, and CDUS-IJV. The latter includes dynamic head-rotation maneuvers (“head-turning test”) to evaluate posture-dependent IJVS, especially at the J3 segment, with systematic evaluation of J1-J3 segments regarding vessel caliber, flow velocity, volume, waveform, and valvular function. In cases suggestive of intracranial hypertension (e.g., headache or visual disturbances), neuro-ophthalmological assessments are performed, including visual acuity and field testing, funduscopy, OCT to quantify RNFL thickness, and ocular ultrasonography to measure ONSD. Non-invasive ICP monitoring or lumbar puncture for direct ICP measurement is performed when indicated. For patients meeting interventional criteria, DSA is conducted, followed by individualized treatment strategies such as venous sinus stenting, internal jugular vein decompression, optic nerve sheath fenestration, acetazolamide therapy, anticoagulation, weight reduction, and symptomatic management. Abbreviations: CVOI, cerebral venous outflow insufficiency; CTA, computed tomography angiography; MRA, magnetic resonance angiography; MRI, magnetic resonance imaging; CE-MRI, contrast-enhanced magnetic resonance imaging; BB-MRI, Black-Blood MRI; CE-CTV, contrast-enhanced CT venography; CE-MRV, contrast-enhanced magnetic resonance venography; DSA, digital subtraction angiography; ICP, intracranial pressure; OCT, optical coherence tomography; RNFL, retinal nerve fiber layer; ONSD, optic nerve sheath diameter; IJVS, internal jugular vein stenosis; IJV, internal jugular vein, CDUS-IJV, color Doppler ultrasonography of the internal jugular vein; TCD, Transcranial Doppler; CADU, carotid artery duplex ultrasonography.


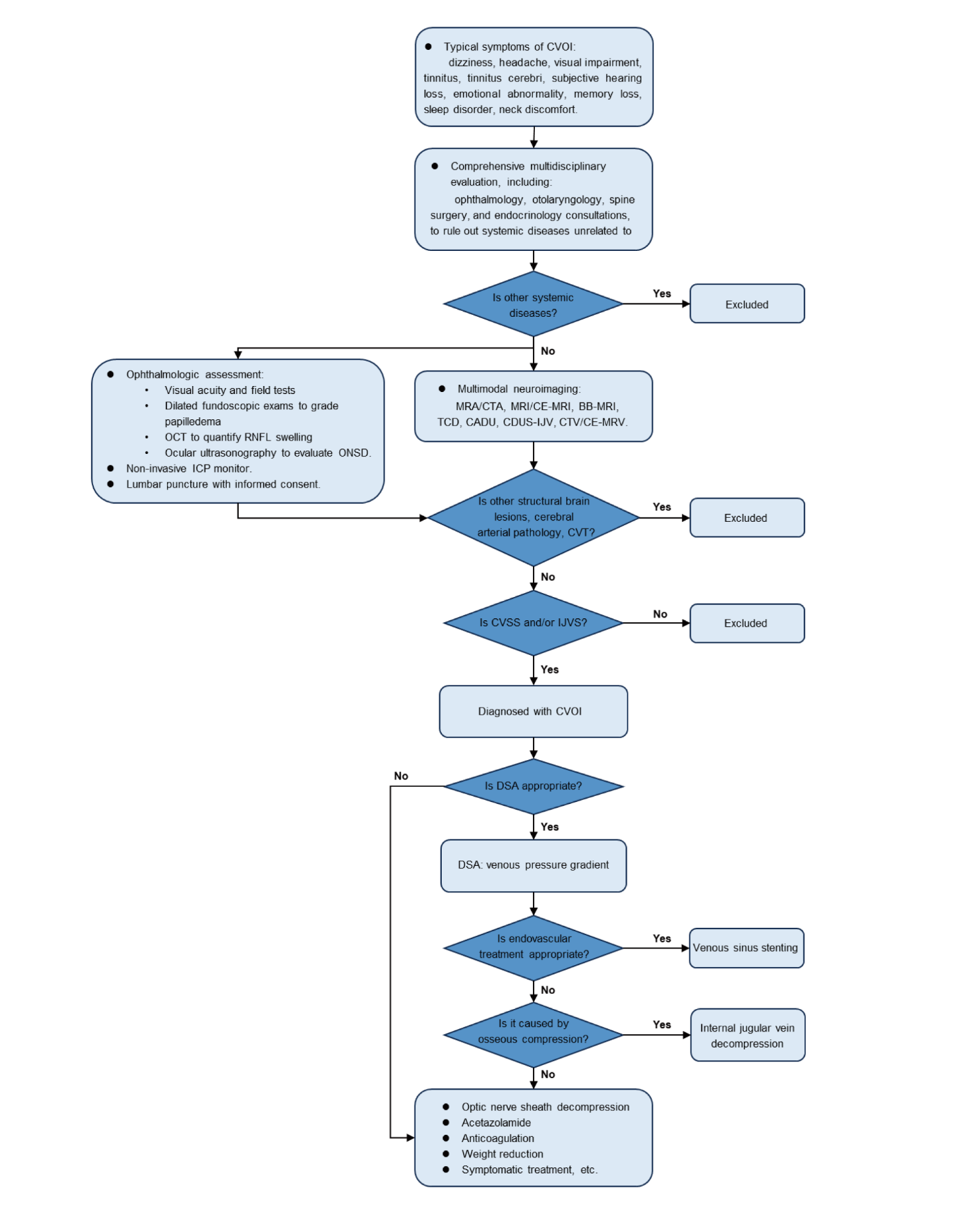

Supplement: Supplementary file 1 — Table S1: Comparison of the caliber of jugular foramen (right vs. left). Table S2: Comparison of the caliber of jugular foramen (female vs. male). Table S3: Comparison of bilateral jugular foramen caliber narrowing rates (right‐type vs. left‐type). Table S4. Comparison of bilateral jugular foramen caliber narrowing rates (females vs. males). Table S5: Demographic data, symptomatic data, and imaging features of CVOI with traditional classification. Table S6: Demographic data, symptomatic data, and imaging features of CV type CVOI. Table S7: Demographic data, symptomatic data, and imaging features of JV type CVOI. Table S8: Demographic data, symptomatic data, and imaging features of CJV type CVOI. Table S9: Demographic and symptomatic data of newly proposed CVOI classification. Figure S1: Determination of optimal thresholds of narrowing rate in bilateral jugular foramen caliber and their predictive performance for imaging‐based classification. (A and B) Histograms showing the distribution of narrowing rates in the bilateral jugular foramen caliber and classification based on optimal cut‐off values. The best cut‐off points determined by the Youden index were 0.203 (A) and 0.395 (B), used to stratify subjects into different narrowing rate groups. (C and D) Curves of Youden index across a range of narrowing rate thresholds. The maximum Youden index indicated the optimal cut‐off values of 0.203 (C) and 0.395 (D), respectively. (E and F) ROC curves evaluating the predictive performance of narrowing rate thresholds (0.203 and 0.395) for imaging‐based classification. Both thresholds demonstrated excellent discrimination, with AUC values of 1.000. ROC, receiver operating characteristic; AUC, area under the curve. Figure S2: Standardized diagnostic workflow for patients with CVOI. This flowchart outlines the standardized diagnostic approach employed in this study for patients with suspected CVOI‐related symptoms. The workflow begins with a multidisciplinary clinical evaluation [file MCO2-7-e70609-s001.docx]
